# On The Retrograde Transport of RNA-Loaded Lipid Nanoparticles Designed for Brain Delivery

**DOI:** 10.1021/acsnanoscienceau.5c00042

**Published:** 2025-08-22

**Authors:** Stefania Mamberti, Cristiano Pesce, Greta Avancini, Gonna Somu Naidu, Govinda Reddy Kundoor, Corinne Portioli, Dan Peer, Paolo Decuzzi, Roberto Palomba

**Affiliations:** † Laboratory of Nanotechnology for Precision Medicine, 121451 Italian Institute of Technology, Genoa 16163, Italy; ‡ Department of Pharmaceutical and Pharmacological Sciences, University of Padua, Padua 35131, Italy; § Laboratory of Precision Nanomedicine, Shmunis School of Biomedicine and Cancer Research, 26745 Tel Aviv University, Tel Aviv-Yafo 69978, Israel; ∥ Department of Materials Sciences and Engineering, Tel Aviv University, Tel Aviv-Yafo 69978, Israel; ⊥ Center for Nanoscience and Nanotechnology, Tel Aviv University, Tel Aviv-Yafo 69978, Israel; # Cancer Biology Research Center, Tel Aviv University, Tel Aviv-Yafo 69978, Israel; ¶ School of Medicine/Division of Oncology, Center for Clinical Sciences Research, Stanford University, Stanford, California 94305, United States

**Keywords:** LNP, RNA, primary cortical neurons, axonal retrograde transport, ionizable lipids, local transfection, microfluidic chip

## Abstract

Lipid nanoparticles (LNP) have been extensively studied for their ability to encapsulate and protect RNA molecules from degradation. More recently, a few studies have begun to explore their applications as carriers for brain drug delivery via various administration routes. Nose-to-brain delivery represents a promising alternative to both invasive local injections and systemic administration, offering the possibility to bypass the blood–brain barrier and directly access the brain, achieve rapid absorption, reduce systemic exposure, and allow for ease of administration. In order to evaluate the viability of this alternative route, it is essential to acquire a better understanding of the intraneuronal mass transport of LNP, particularly in terms of how effectively and efficiently they deliver their payloads from the periphery to neuronal cell bodies. However, most previous studies have focused primarily on the delivery vector itself rather than on the fate of the transported cargo. In this study, we investigate the retrograde trafficking of nucleic acid-loaded LNP in primary cortical neurons, focusing on the transport of both the particle and the payload. Three distinct LNP were formulated to characterize different aspects of their interaction with the cells, with the major LNP player of this study containing a red-fluorescent Rhodamine B-tagged lipid and a green fluorescently FAM-tagged RNA. Flow cytometry was used to document LNP uptake by primary cortical neurons over time. Additionally, confocal microscopy was then used to investigate the colocalization of LNP and RNA after a conventional 2D culture treatment. As a final step, a compartmentalized chip that separates the somal and the axonal regions of cortical neurons was used to study the intraneuronal dynamics of LNP and their cargo. In this second setup, LNP were selectively administered at the axonal compartment, and the fluorescent signals from the vector (red) and the payload (green) were imaged through time-lapse microscopy. The progressive accumulation of RNA found at cellular bodies also in the absence of the red signal suggested an efficient retrograde transport of the LNP payload toward the soma. Comprehensively, this work demonstrates that primary cortical neurons are capable of efficiently uptaking LNP and of intracellularly transporting both LNP and their RNA cargo. Interestingly, a different colocalization trend (LNP–RNA) emerged depending on the followed setup. Localized axonal transfection appeared to favor dissociation of RNA from the LNP and subsequent accumulation at the soma. Overall, our work provides a fundamental in vitro proof of concept of the RNA delivery to the cellular bodies of primary cortical neurons via the retrograde transport of LNP vectors administered at the axonal termini. This finding, together with the image-analysis-based quantification of the RNA accumulation described in our work, paves the way for future studies aimed at designing lipid-based nanoparticles for RNA therapeutic delivery to the brain via peripheral administration.

## Graphical abstract

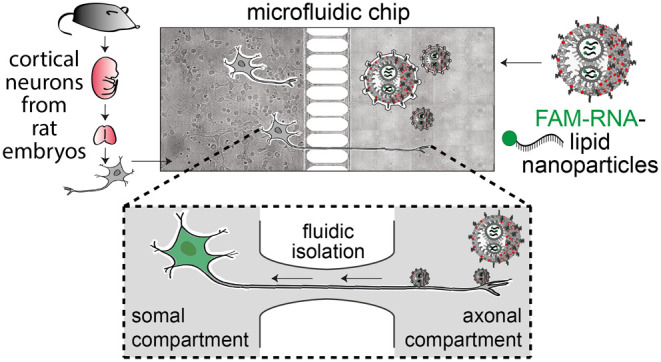

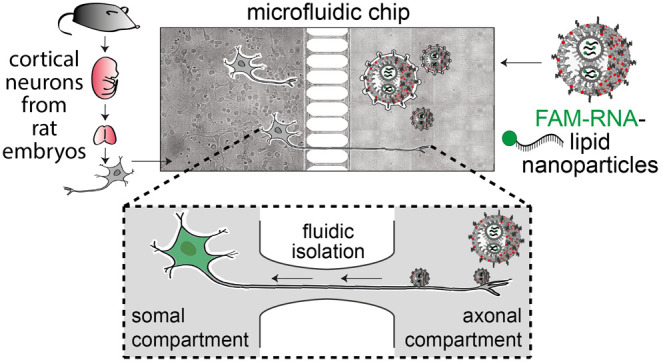

## Introduction

In recent decades, lipid nanoparticles (LNP) have become exponentially popular as drug delivery systems.^1^ Due to their ability to entrap nucleic acids and protect them from degradation, they are being used for the treatment of a wide range of diseases.^2–4^ Additionally, LNP are highly biocompatible, biodegradable, structurally flexible, easily engulfed by various cells, and present low immunogenicity and toxicity, hence gaining increasing approval in the clinic.^5^ In the context of brain delivery, although LNP coated with apolipoprotein E (ApoE), transferrin, and other molecules have shown some potential to cross the blood–brain barrier,^6^ their systemic administration is typically impaired by the common limitations of most nanomedicines, which undergo unspecific sequestration in the mononuclear phagocyte system and off-target accumulation.^7,8^ On the other hand, local administration improves brain-targeting but involves invasive procedures such as intracranial or intrathecal injections.^9,10^

An alternative approach is the so-called nose-to-brain delivery, which exploits the innervation of the nasal mucosa by the olfactory and trigeminal nerves as a bridge to directly access the brain from outside the central nervous system.^11,12^ This route is noninvasive, generally well tolerated, and minimizes drug clearance from the bloodstream. Drugs delivered at the upper nasal space can cross the olfactory epithelium in between the tight cell junctions or through the epithelial cells (paracellularly or transcellularly) or directly inside the neuronal axons (intracellularly), since the long cilia of the olfactory nerve endings are extended until the mucosa interface with the environment. Drugs deposited at the upper anterior and lower segments of the nasal space will need to cross the epithelium either para- or transcellularly first, before reaching the trigeminal nerve, whose endings are not projected above the epithelial surface.^11^ Following the uptake by the olfactory and trigeminal nerves, drug delivery to the actual biological target relies on the intracellular transport from the axonal termini at the nasal mucosa interface to deeper regions of the brain.^11,12^ This transport, from the periphery (axons) to the cellular body (soma) of neurons is known as retrograde axonal transport, in contrast to the anterograde transport, which follows the opposite direction.^13–16^ These transport mechanisms rely on cytoskeletal rails (i.e., microtubules, neurofilaments, and actin filaments^17^) onto which molecular motors (i.e., dynein, kinesin, myosin^18^) transport various cargoes often packaged into vesicles (e.g., axonal membrane proteins^19^). Currently, several works have been published on the retrograde axonal transport of lipidic formulations loaded with different drugs for nose-to-brain delivery.^12,20^ Most of the investigations using LNP or other particles focus on the fate of the vector, not providing information on the transported molecule.^21–25^

In this work, we investigate the axonal retrograde transport of LNP carrying RNA molecules, focusing both on the carrier and on the payload by virtue of a double fluorescence label, and we compare the outcome of a regular treatment on entire neurons (cell bodies and axons) and of a local treatment on confined axons. To run this study, three different LNP formulations were engineered (Empty LNP, LNP with a fluorescent Rhodamine B tracer on the lipid moiety: RhB-LNP, and RhB-LNP loaded with a carboxyfluorescein fluorescent dye conjugated to the RNA: FAM-RNA-LNP), all three containing a specific ionizable amino lipid designed to promote the efficiency of the transfection (Lipid 15 in^26^). Cortical neurons were isolated, and upon validation of LNP biocompatibility, particle uptake was tested via flow cytometry. The colocalization of LNP and RNA was measured by using confocal microscopy until 8 h post treatment. Cortical neurons were additionally cultured in a compartmentalized microfluidic chip, which allowed for the physical separation of the axons and somas. FAM-RNA-LNP were used for local transfection in the regional area of the axons. Under these conditions, mimicking those of nose-to-brain delivery, the transport of LNP and of the RNA were observed using time-lapse microscopy.

## Results

### Synthesis of FAM-RNA-Lipid Nanoparticles

Three lipid nanoparticle (LNP) formulations with distinct compositions were produced and characterized, namely, particles carrying no cargo, Empty LNP; particles incorporating the red-fluorescent lipid 18:1 lissamine rhodamine B phycoerythrin PE lipid (RhB-lipid)—RhB-LNP; and particles carrying both the RhB-lipid and a scrambled RNA labeled with the green-fluorescent molecule FAM—FAM-RNA-LNP. The material for LNP production and the molar ratios of the lipid mixtures of all three formulations are reported in Supporting Information Tables S1 and S2, respectively.

All LNP formulations were synthesized using a benchtop microfluidic-based device to obtain particles with a uniform size and highly reproducible features across different batches. Figure 1A shows a schematic representation of the synthesis process for the FAM-RNA-LNP, where an aqueous solution and an organic one are mixed within a microfluidic cartridge at a predetermined total flow rate of 8 mL/min, with a 1:3 aqueous/organic flow rate ratio.^27,28^ The aqueous solution contained FAM-RNA (240 μg/mL), whereas the organic solution contained RhB-lipid, cholesterol, ionizable lipid (Lipid 15), helper lipid (dioleoylphosphatidylcholine (DOPC)), and PEGylated lipid (DMG-PEG) dissolved in ethanol. Empty LNP and the bare RhB-LNP were produced following the same protocol with slight modifications in the initial compositions of the two solutions, based on previous studies which successfully used Empty LNP as a control system versus RNA-bearing LNP.^29–31^

**Figure 1:**
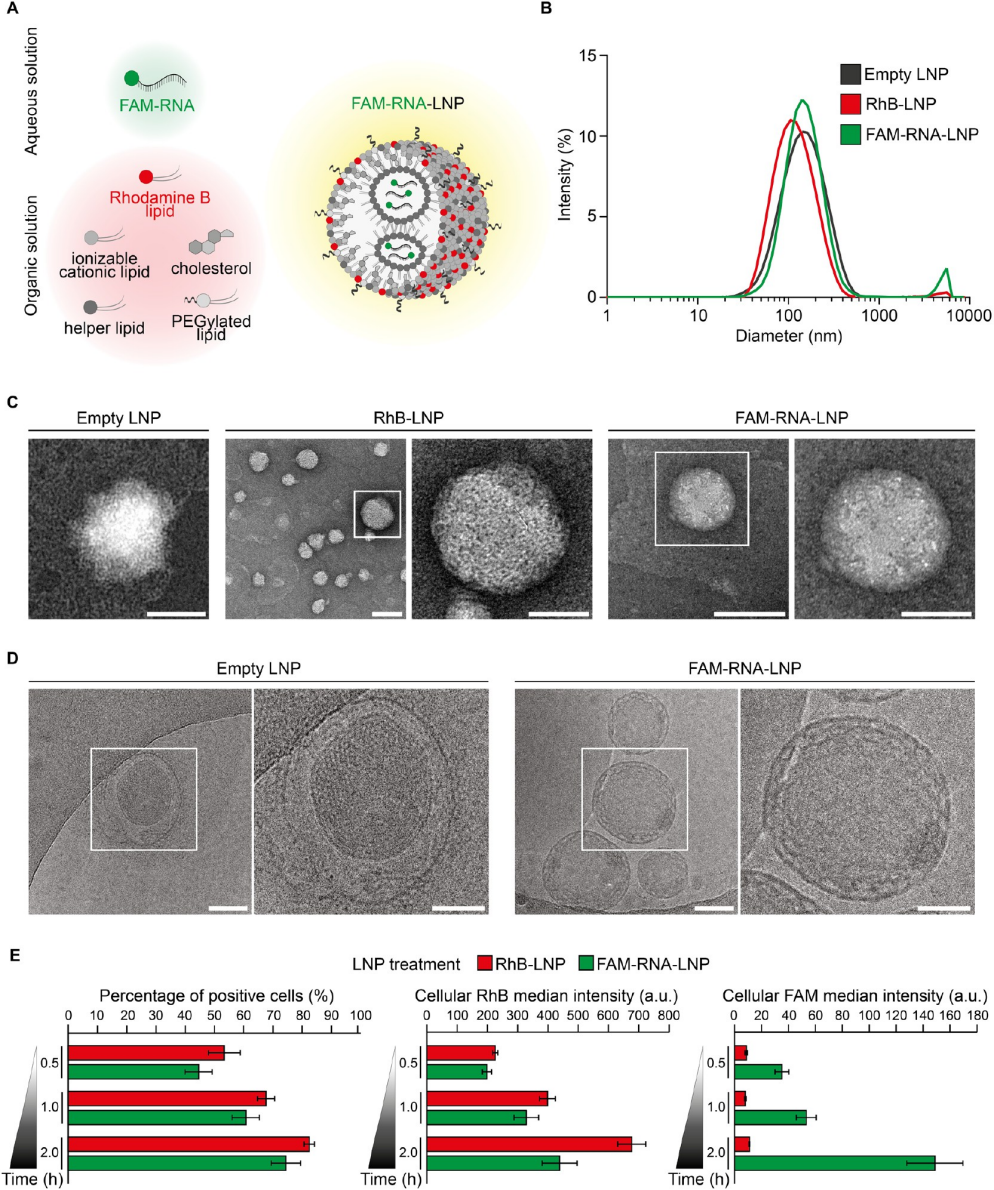
LNP synthesis and physicochemical characterization. (A) Schematic for the synthesis of FAM-RNA-LNP via microfluidic mixing. An aqueous solution containing FAM-RNA was mixed with an organic solution containing Rhodamine B-lipids (18:1 Liss Rhod PE lipid), cholesterol, ionizable lipids (Lipid 15), helper lipids (DOPC), and PEGylated lipids (DMG-PEG) dissolved in ethanol, within a microfluidic cartridge at a total flow rate of 8 mL/min with a 1:3 aqueous/organic flow rate ratio. (B) Intensity profile for the hydrodynamic diameters of the three formulations (Empty LNP, RhB-LNP, FAM-RNA-LNP) via dynamic light scattering characterization (DLS). Results of the DLS measurements are given for Empty LNP, RhB-LNP, FAM-RNA-LNP, respectively, (average of *n* = 3 replicates ± standard deviation): size (nm): 111.67 ± 40.33, 123.50 ± 29.29, 160.10 ± 11.67; polydispersity index, PdI: 0.23 ± 0.01, 0.20 ± 0.02, 0.29 ± 0.08; surface electrostatic ζ-potential (mV): 19.17 ± 6.89, 12.69 ± 1.02, 8.77 ± 2.11. (C,D) TEM and cryo-EM representative images of the three lipid nanoparticle formulations, respectively. The magnified inset represents the area cropped from the white square. Scale bar: 100 nm in the main images, 50 nm in the magnified insets. (E) Percentage of cells associated with either RhB-LNP or FAM-RNA-LNP, via flow cytometry considering the RhB-lipid signal (left). Cellular median RhB (center) and FAM (right) intensities are shown for the fraction of the neuronal population that was considered positive to LNP association (red bars: neurons incubated with RhB-LNP; green bars: neurons incubated with FAM-RNA-LNP).

All three were formulated with an ionizable amino lipid containing an ethanolamine linker (Lipid 15). This lipid was previously shown in vivo to allow efficient RNA transfection dictated by RNA endosomal escape to which Lipid 15 contributes thanks to the presence of its acid-sensitive linker and hydrophobic tails.^26,32^

Size and surface charge of Empty LNP, RhB-LNP, and FAM-RNA-LNP were characterized via dynamic light scattering (DLS), returning the distribution plots shown in Figure 1B. Hydrodynamic diameters of 111.67 ± 40.33, 123.50 ± 29.29, and 160.10 ± 11.67 nm were measured for Empty LNP, RhB-LNP, and FAM-RNA-LNP, respectively, with values of PdI ranging between 0.20 and 0.29. Empty LNP returned the highest surface electrostatic ζ-potential, being +19.17 ± 6.89 mV, followed by RhB-LNP with a value of +12.69 ± 1.02 mV, and finally FAM-RNA-LNP with +8.77 ± 2.11 mV. It is important to note here that, in the original LNP mixture, FAM-RNA could partially be exposed on the surface, thus affecting the surface electrostatic ζ-potential. Indeed, the moderate positive charge associated with the Empty LNP and RhB-LNP should be ascribed to the ionizable lipid, which, in slightly acidic water, tends to be protonated and therefore positively charged.^33,34^ Notice that with the addition of the negatively charged FAM-RNA, the overall charge of the LNP was reduced. However, the small differences in size and ζ-potential across the three different LNP configurations were all measured to be not significant (Supporting Information Table S3). Transmission and cryo-electron microscopy images of LNP are provided in Figure 1C,D, respectively. LNP appear with a regular spherical shape and a diameter comparable with those of the DLS measurements. The cryo-electron microscopy images of the FAM-RNA-LNP reveal details of the internal structure of these particles. Specifically, a “lace doily”-like structure is observed, with a lipid monolayer surrounding smaller nanovesicles, likely driven by the electrostatic interactions between the ionizable lipid and the anionic RNA cargo. This structural arrangement is consistent with previous findings by other authors.^35^ Freeze-drying studies were additionally performed with the aim of increasing their potential for future applications, proving good stability of the LNP lyophilized with 15% (w/v) trehalose up to 28 days of storage (Supporting Information Figure S1).

### Lipid Nanoparticles Biocompatibility and Association with Primary Cortical Neurons

Cortical neurons were isolated from rat embryos as described in Materials and Methods and seeded in regular 2D culture multiwell plates. First, the viability of cortical neurons incubated with Empty LNP was assessed through an MTT assay. Empty LNP at different amounts, ranging from 0 to 40 μg of total lipid content, were incubated with primary cortical neurons. The cellular metabolic activity was assessed at 2, 24, and 48 h post incubation. As shown in Supporting Information Figure S2, no significant changes were observed at all predetermined time points and tested concentrations. The 5 μg total lipid content, corresponding to a 1× fold dose in Supporting Information Figure S2, was selected as the working dose for all subsequent experiments.

Then, LNP association with primary cortical neurons was assessed via flow cytometry, at 0.5, 1, 2 h post incubation (Figures 1E; Supporting Information Figure S3). For these experiments, fluorescently labeled RhB-LNP and FAM-RNA-LNP (also containing the RhB-lipid) were used. Note that RhB-LNP were here used as a fluorescence control to precisely discriminate the FAM-RNA signal from a possible channel bleed-through of the RhB signal in the FAM-RNA-LNP uptake, while the Empty LNP were used to set the fluorescence threshold. The gating for positive cells was set at the mean intensity of 10^2^ a.u., at which neurons treated with Empty LNP do not show any fluorescence. Supporting Information Figure S3B shows representative results, where the cells treated with Empty LNP do not show any fluorescence above the set threshold, while the intensity distribution shifts beyond it in the red channel for both RhB- and FAM-RNA-LNP and in the green channel for FAM-RNA-LNP. The data collected in Figure 1E (left) shows the percentage of neurons associated with RhB-LNP (red bars) and FAM-RNA-LNP (green bars), at predetermined time points, namely, 0.5, 1, and 2 h of incubation. As expected, the percentage of cells associated with LNP increases with the incubation time, varying from nearly 50% at 30 min up to over 80% at 2 h post incubation. Indeed, the longer the incubation time is, the longer is the likelihood that LNP would encounter a cortical neuron and either adhere to its surface or be engulfed. No statistically significant difference in neuronal association between RhB-LNP and FAM-RNA-LNP was detected, which is aligned with the negligible difference in morphological and physicochemical properties between the two carriers (Figure 1B). Furthermore, Figure 1E (center) and (right) document the change over time in red-fluorescent (PE intensity) and green-fluorescent (Fluorescein Isothiocyanate (FITC) intensity) signals, for the fraction of cells associated with LNP. Specifically, in Figure 1E (center), the PE intensity, due to the RhB-lipid dispersed within the LNP structure, was observed to increase progressively over time from 226.11 ± 8.49 a.u. at 30 min up to 676.33 ± 46.06 a.u. at 2 h for RhB-LNP and from 198.67 ± 15.24 a.u. up to 438.56 ± 57.67 a.u. for FAM-RNA-LNP. In Figure 1E (right), the FITC signal from FAM-RNA packed within the LNP showed an increase only for the neurons exposed to FAM-RNA-LNP from 35.11 ± 5.17 a.u. at 30 min up to 148.78 ± 20.81 a.u. at 2 h. Indeed, the RhB-LNP returned a constant, basal signal, as they lack the FAM-RNA and the related green coloration. The growing cellular median intensity measured for PE and FITC over time is related to the increase in the number of LNP associated with primary neurons. Cumulatively, these data indicate that both the number of cells positive for LNP (Figure 1E, left) and the number of LNP per cell (Figure 1E, center and right) increase over time for all tested LNP formulations.

### Colocalization of LNP and RNA after Treating Neurons Cultured in a Regular 2D Culture Setup

To gain more insights into the topography of the signals from LNP and from RNA, primary cortical neurons were incubated with FAM-RNA-LNP and imaged via fluorescent confocal microscopy at predetermined time points until 8 h post incubation. Representative microscopy images of this analysis are shown in Figure 2A. The red and green signals are associated with the LNP carrying RhB-lipid and FAM-RNA, respectively, while the blue and magenta signals are associated with the nucleus (DAPI staining) and the cytoskeleton (neurofilament immunostaining) of the neurons, respectively. At 0.5 h of incubation with LNP, a red punctuated signal given by the RhB-lipid already overlays with the neurofilament-stained cortical neurons, proving the cellular association of LNP. This is especially clear on the axonal branches after 1 h of incubation. The number of dots increases with time, representing an increase in the level of LNP associated with neurons. Internalization goes further from 2 to 8 h of incubation, with an increased fluorescence intensity in the red channel (LNP). The accumulation of green FAM-RNA, although detectable at 0.5 h, appears more subtle until 1 h after incubation with LNP.

**Figure 2:**
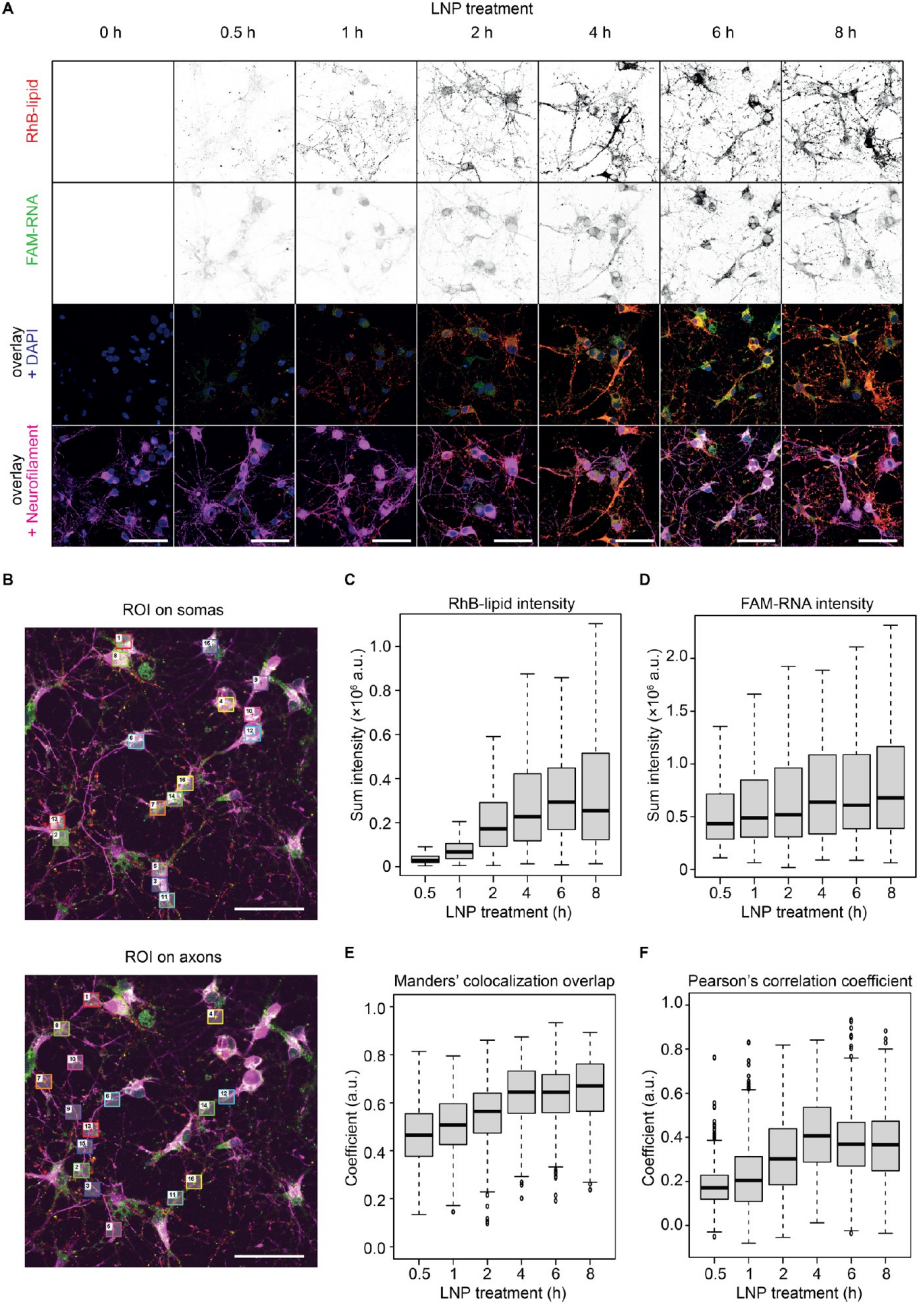
Confocal microscopy analysis of LNP neuronal uptake. (A) Representative confocal microscopy images of cortical neurons treated with FAM-RNA-LNP at predetermined time points (red: RhB-lipid; green: FAM-RNA; magenta: neurofilament; blue: nucleus). Montages are shown of a single *z*-plane from image stacks, with the single channels for RhB-lipid and FAM-RNA in gray and their overlay with nuclei and neurofilament in colors. Scale bar: 50 μm. (B) Representative regions of interest (ROI) selected on the soma (top) and axons (bottom) of neurons. ROI were randomly selected within the neuronal body delineated by the neurofilament immunostaining. The image is from a sample of cortical neurons treated with FAM-RNA-LNP for 6 h. Scale bar: 50 μm. (C,D). Quantification of RhB-lipid and FAM-RNA fluorescence intensity within the randomly selected ROI. (E). Manders’ colocalization coefficient for the randomly selected ROI over time. (F). Pearson’s correlation coefficient for the randomly selected ROI over time.

The FAM-RNA signal intensifies from 2 h until 8 h, a range in which an increasing number of cellular bodies and axonal branches appear as yellow in the overlay images due to the overlap between the red and the green signals (Figure 2A, bottom).

To extract quantitative data, different regions of interest (ROI) were randomly identified both in the soma and along the axons of the cortical neurons, as shown in the image of neurons treated with FAM-RNA-LNP for 6 h in Figure 2B. Note that the neurons coincide with the neurofilament positively stained areas (magenta signal). Within all of the ROI, the RhB-lipid and FAM-RNA signal intensities were quantified and plotted against incubation time in Figure 2C,D, respectively. These quantitative analyses confirmed an increase in intensity over time for both signals, thus indicating a progressive LNP engulfment followed by a parallel increase in the FAM-RNA signal. Consistently with the images in Figure 2A, the RhB-lipid intensity gets a steeper increase at 2 h of LNP incubation compared to the earlier time points and grows further until 8 h (Figure 2C), while the FAM-RNA signal intensifies more gradually over time (Figure 2D). Both reach a sort of plateau between 6 and 8 h of incubation, again consistently with the visual inspection that shows more and more cells being fully filled with nanoparticles. To further investigate the topography of the signals from the fluorescent components of FAM-RNA-LNP, the colocalization of RhB-lipids and FAM-RNA was assessed within the neurons over time using Manders’ and Pearson’s analyses. The Manders’ colocalization coefficient indicates the overlap between two signals and ranges between 0 and 1 (0: no colocalization, 1: perfect colocalization). The Pearson’s correlation coefficient measures the linear correlation between two sets of data with a coefficient ranging between −1 and 1 (1: perfect linear correlation, −1: perfect inverse linear correlation, 0: no correlation). Therefore, referring to the same ROI, the Manders’ and the Pearson’s correlation coefficients were calculated over the three most central *z*-planes of the confocal image stacks and plotted against the incubation time, in Figure 2E,F, respectively. Both the pure colocalization (expressed by Manders’ coefficient in Figure 2E) and the linear correlation (expressed by Pearson’s coefficient in Figure 2F) of the two signals show an increase from 0.5 to 4 h of incubation with LNP. Consistently with the microscopy images in Figure 2A, both coefficients reach the highest values and get closer to a plateau between 4 and 8 h of treatment. In more detail, Manders’ coefficient indicating the colocalization of two signals reaches average values larger than 0.6 at 4 h after incubation and for the later time points, suggesting LNP and FAM-RNA to be mainly present in the same areas (Figure 2E). Pearson’s coefficient grows from ∼0.2 at 0.5 h of LNP incubation up to ∼0.4 at 4 h and later time points, meaning that the variation in intensity of the two signals becomes more correlated over time, indicating the concomitant accumulation of RhB-lipid and FAM-RNA within the neurons (Figure 2F). In summary, from these results we can understand that the particles and the RNA undergo the same fate until 8 h after treatment (intended as a cumulative phenomenon being based on the calculation of an average). In Supporting Information Figure S4, both Manders’ and Pearson’s coefficient analyses are shown separately for the ROI in the soma and the ROI along the axons of the cortical neurons.

### Primary Cortical Neurons Cultured into Compartmentalized Microfluidic Chips

Cortical neurons were seeded in a microfluidic chip, including a somal and an axonal compartment, separated by multiple parallel 150 μm-long microgrooves (Figure 3A). The microchip system allows to perform fluidically isolated regional treatments of primary neurons and was extensively validated in previous studies.^16,36–39^ The neuronal culture was initially seeded on the somal compartment and then imaged through wide-field microscopy at predetermined days to check for cell viability, the development of the neuronal network, and the protrusion of axon filaments from within the microgrooves (Figure 3B). During the first few days, neurons started interacting with one another through their axonal branches. Between days 3 and 4, some of the axons grew along the microgrooves. On day 5, axons reached the other side of the microgrooves and started expanding in the axonal compartment. Around day 11, the axons reached full maturity with the highest extension and network complexity, which was maintained with only minimal changes thereafter. The length of those axons reaching the axonal compartment was measured over time between days 4 and 7. The plot in Figure 3C shows the variation in length of the axons (*n* = 10) growing from the somal compartment toward the axonal one and reaching an average length of ca. 500 μm at day 7. A considerable variation in length was observed at day 7, with the extremes being around 200 μm and 850 μm long axons, demonstrating the intrinsic variability of the problem at hand. An image of a single axon growing over time is reported on the right side of Figure 3C, where a colored legend is used to identify the axonal length at different time points.

**Figure 3:**
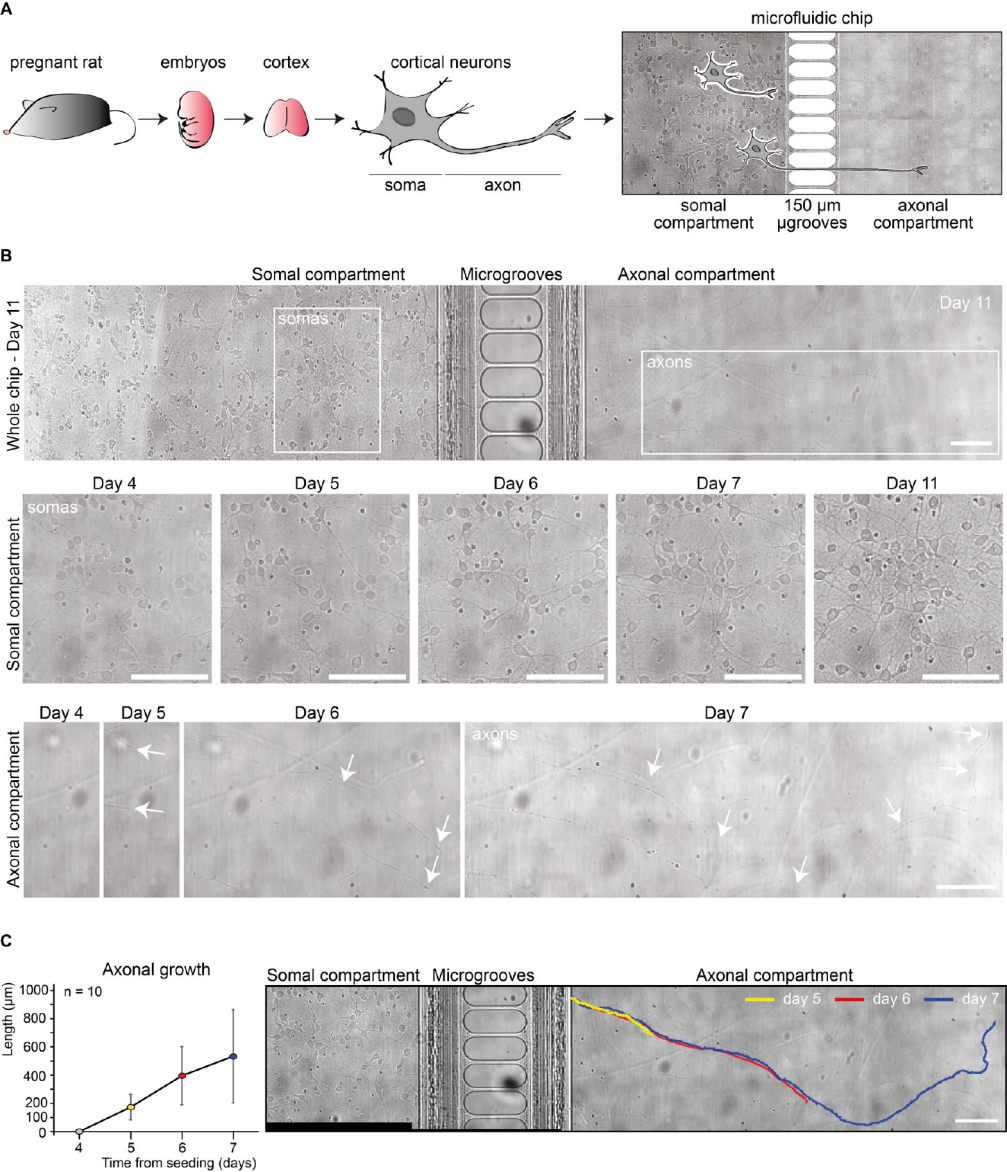
Primary neuronal culture and axonal growth in compartmentalized microfluidic chips. (A) Experimental schematic of cortical neurons’ isolation from rat embryos and seeding in the microfluidic chip. Isolated neurons were plated on one side (somal compartment) of a microfluidic chip and allowed to extend their axons through the 150 μm-long microgrooves bridging to the other side (axonal compartment). (B) Representative widefield microscopy brightfield images of the neuronal culture between days 4 and 11 post seeding. Arrows identify the extremities of neuronal axons. Scale bar: 100 μm. (C) Analysis of axonal growth between days 4 and 7 (left). Length of a representative axon at day 5 (yellow), day 6 (red), and day 7 (blue) (right). The extent of axonal growth from the end of the microgrooves inside the axonal compartment was measured on widefield microscopy brightfield images. Note that, from day 8 on, it was no longer possible to perform unbiased measurements of single axons due to the complex and intricate network formed by axons originating from different somas. Scale bar: 100 μm.

Once the neuronal culture in the microfluidic chip was established, as reported above and in the Materials and Methods section, LNP axonal uptake and transport were investigated. As schematically depicted in Figure 4A, fully grown cortical neurons, between days 7 and 15 post seeding, were exposed to FAM-RNA-LNP on the axonal compartment. Figure 4B shows representative images of the whole chip after 5 and 8 h of incubation. To have an overview of signal fluctuation over time, the mean intensity and the standard deviation of both signals in the somal compartment were extracted and normalized with respect to time 0 in order to quantify the signal over time (Figure 4C, left and right). Both RhB-lipid and FAM-RNA mean signals increase over time, indicating that the LNP are being trafficked into the somal compartment. Moreover, the standard deviation of the two signals also increases, suggesting that the intensity does not increase homogeneously in the compartment but rather some areas accumulate more signal than others, and hence the signal of those areas progressively deviates more from the background. Indeed, cropped sections of the somal compartment in Figure 4D show the single cellular bodies of cortical neurons lighting up from the background in both channels, each with a brighter intensity at subsequent time points.

**Figure 4:**
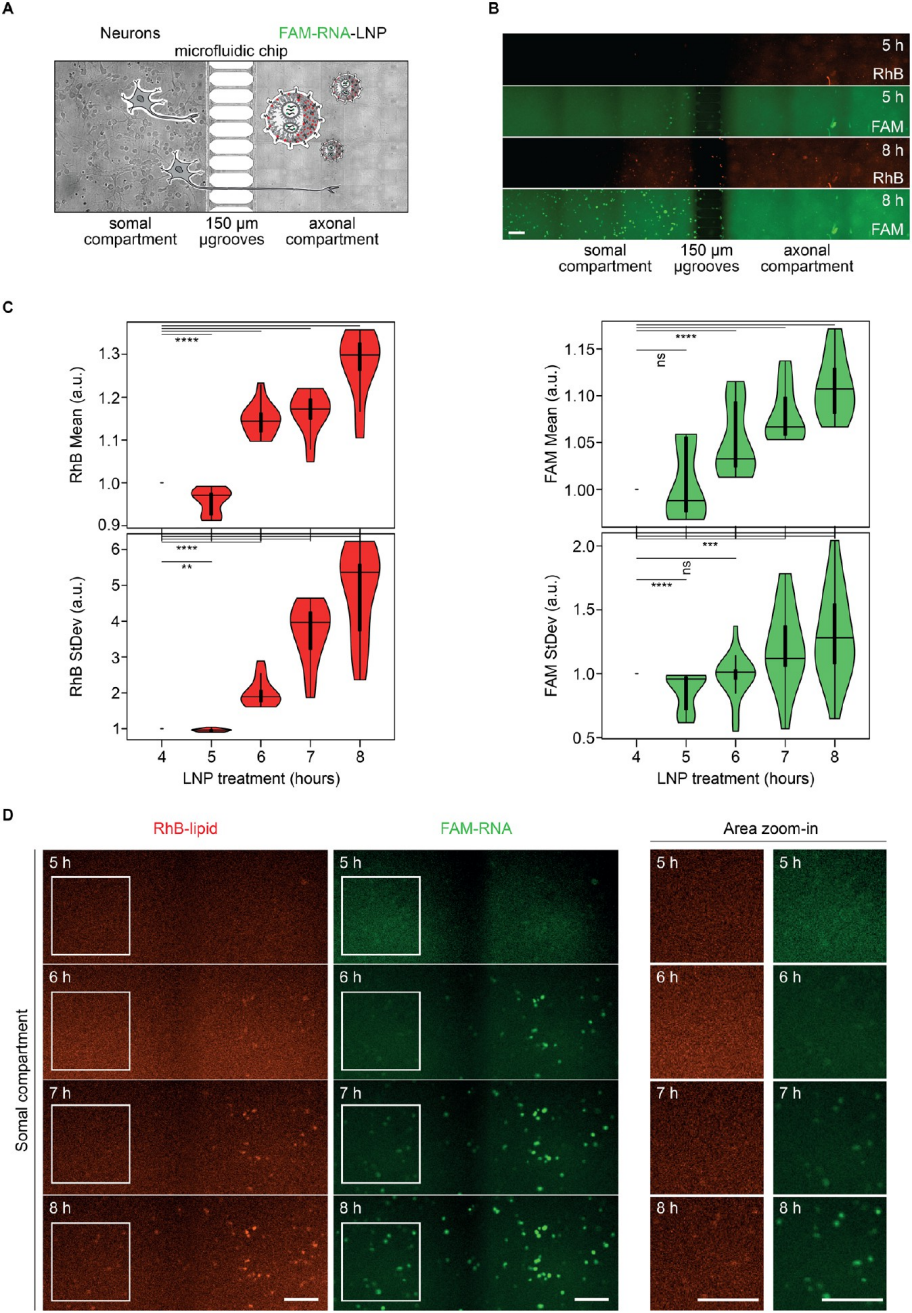
Retrograde transport of LNP in cortical neurons: whole somal compartment analysis. (A) Schematic of FAM-RNA-LNP treatment on cortical neurons within the microfluidic chip. (B) Representative time-lapse microscopy images of the whole chip upon 5 and 8 h of LNP treatment, in the RhB-lipids (red) and FAM-RNA (green) channels. Scale bar: 100 μm. (C) Analysis of LNP increase in the somal compartment over time: normalized mean intensity (top) and standard deviation (bottom) of the fluorescence signal for RhB-lipid (left) and FAM-RNA (right). (D) Representative time-lapse microscopy images of LNP accumulation over time in the somal compartment. An area of the somal compartment is shown for both RhB-lipid (red) and FAM-RNA (green) channels at all time points (left). Magnification of the white squared regions from the left panels (right). Scale bar: 100 μm.

On the right side of Figure 4D, the magnified insets show the peripheral area of the soma compartment (the most distant from the microgrooves, delimited by the white square in the left images). The insets allow to appreciate the faster accumulation of FAM-RNA with respect to RhB-lipids in the somas of cortical neurons. Indeed, single somas gradually light up and emerge from the background in the green channel, while the same peripheral somas are difficult to discriminate from the background in the red channel. These results suggest that some of the RNA is transported intracellularly, independently from the vector.

In addition, single cell analyses were also conducted to follow the RNA signal increase over time (see Supporting Information Figure S5 for the image analysis pipeline). For this purpose, single neurons were identified and masked in the somal compartment of the microfluidic chip through a threshold based on the FAM signal observed at the latest time point, namely, 8 h (Figure 5A). While no neuron was positive at the initial time point (4 h incubation), in the imaged area more than 500 neurons became positive 8 h post incubation (Figure 5B, top; see Supporting Information Figure S6 for an overview of the timeline of FAM-RNA cargo release in single neurons). To highlight the RNA diffusion and accumulation inside the somas, two other parameters were also considered: the sum of the green-fluorescent signal found in each soma and the total area covered by the same signal within the segmented somas (Figure 5B, center and bottom, respectively; see Supporting Information Figure S7 for details on the single-cell quantification).

**Figure 5:**
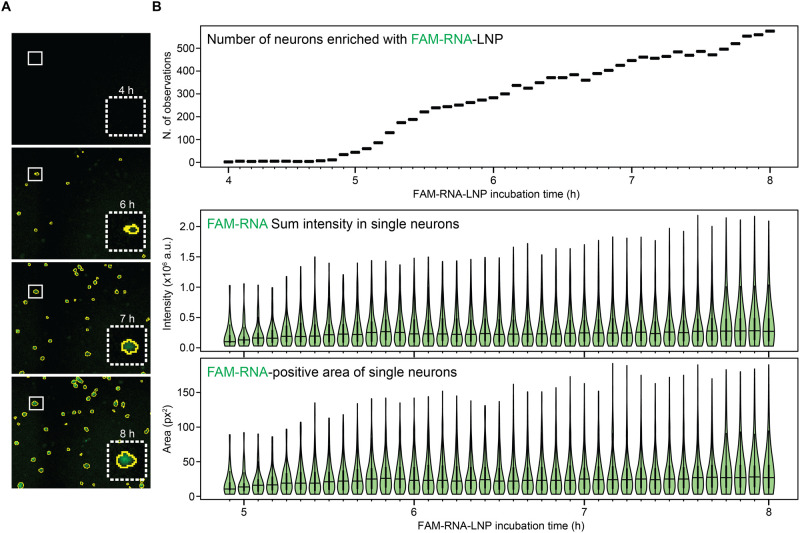
Retrograde transport of FAM-RNA-LNP in cortical neurons: single neuron analysis. (A) Representative time-lapse microscopy images showing the selection of FAM-RNA-positive neurons based on image analysis. (B) Analysis of the FAM-RNA increase in the soma of single neurons over time from time-lapse microscopy images. Number of positive neurons (top), cellular FAM sum intensity (center), relative FAM-positive area (bottom) variation over time.

The plots show that both parameters increase over time. Collectively, these data indicate that an increasing number of neurons became positive for FAM-RNA over time and their somas continued to accumulate RNA, resulting in a progressively stronger FAM signal and a gradually larger cellular area occupied by FAM-RNA, indicating that a route of transfection involving the passage of RNA through the axon might be particularly efficient in favoring the specific accumulation of the nucleic acid at the level of the soma.

## Discussion

In this study, the authors investigated the internalization and retrograde axonal transport of RNA-loaded LNP in primary cortical neurons, focusing on their accumulation in spatially isolated somas. Empty LNP, RhB-LNP, and FAM-RNA-LNP were prepared via a microfluidic system, displaying a consistent hydrodynamic diameter, polydispersity index, and surface charge. Beyond confirming LNP biocompatibility, the association rate of LNP with primary cortical neurons was analyzed using high-throughput single-cell analysis flow cytometry. The percentage of positive cells ranged from 50% at 30 min up to 80% within the 2 h of incubation.

LNP internalization was further validated through neurofilament immunostaining and confocal microscopy. A previous study with polystyrene nanoparticles carrying no payload observed a consistent intracellular increase at 1.5 h and saturation at 3 h of somatic treatment,^23^ in line with the steeper increase of RhB-lipid intensity that we observed at 2 h of LNP incubation and the relative saturation starting at 4–6 h. Our results also highlighted that with a treatment on the whole neuron, the LNP components RhB-lipids and FAM-RNA colocalize until 8 h post treatment.

In order to analyze LNP uptake and the retrograde transport to the neuronal soma of both LNP and their payload, primary neurons were cultured in a microfluidic chip. This specific chip allows one to separate the soma from the axons and to perform the treatment on the latter only. In line with other authors’ studies, the accumulation of nanoparticles was retrieved not earlier than ca. 4 h and increased evidently ca. 6 h after axonal incubation.^22,23,25,40^ More specifically, the cited studies and most of the existing literature focus on the axonal displacement of empty particles of other materials: gold,^25,40^ polystyrene,^23^ or chitosan,^22^ with no mention on the fate of a putative payload. With the aim to investigate RNA fate, we followed its signal by using time-lapse microscopy and revealed a differential intracellular localization of the vector (LNP) and of the payload (RNA), conversely with the result obtained in the regular 2D culture. In summary, with a regular whole-cell transfection in a conventional 2D culture of neurons, LNP can directly access the cellular bodies, which eventually return high fluorescence intensity levels but still show colocalization of the RhB-lipid and FAM-RNA signal until 8 h of treatment. By performing, instead, the same transfection only on the exposed surface of axons, the LNP and RNA follow different fates, starting from earlier time points. RNA travels toward the soma also in the absence of the vector, while integer LNP and potentially the residual lipids of those nanoparticles that released their payloads are transported toward the soma in a slower fashion. The progressive enrichment of FAM-RNA in somas suggests a robust and dominant RNA release from the retrograde-transported LNP. Thus, somas continued to accumulate FAM-RNA, as indicated by increasing signal intensity and cell area over time, signifying successful retrograde RNA transport along axons to somas. The different outcome of the same experiment performed as a regular transfection and as a local axonal transfection might be depending on a different availability of endosomes at the axon site and at the soma site or on the different mechanism of function of the same organelles in two distinct areas of neurons. These findings are particularly important considering the potential application of LNP presenting an efficient ionizable lipid (as in our case Lipid 15) in nose-to-brain delivery and in the comparison of this route of administration to the local one. The increased and specific RNA accumulation in the cell bodies of cortical neurons locally treated with LNP at their axonal side would suggest that LNP with ionizable lipids could be particularly suitable for designing nucleic acid-based nose-to-brain therapies.

## Conclusions

The presented work compared the outcome of LNP transfection when operated in a regular 2D culture of primary cortical neurons and when operated solely in their axonal compartment. While in the first setup, colocalization of the vector and the payload was present at least until 8 h after administration, tracking RNA and LNP when administered directly and solely on axons returned very different results. More specifically, RNA was clearly detectable in the soma, even in the absence of the signal used to mark LNP. The lack of colocalization between the two signals under this setup possibly indicates that following LNP internalization, RNA could travel within the axon also after being deprived of the LNP envelope. This work hence presents an in vitro, primary-culture-based model specifically designed to test nucleic acids’ retrograde transport and to understand the efficiency of their transfection, when the treatment with their vectors (LNP or any other particles) is specifically operated on axons. This kind of test could be precious in the preclinical evaluation of nucleic acid-based nose-to-brain therapeutics, which requires the integration of different in vitro, ex vivo, and in vivo approaches to adequately recapitulate human body conditions.^41^ In this work, the physical stability of the system was confirmed upon lyophilization, providing strong foundations for the future development of nanoformulations for intranasal administration.^42–45^ To enhance preclinical translation, the LNP formulation could be optimized for mucosal delivery by including specific surface modifications, and LNP stability could be further assessed in vivo within the nasal mucosa. In future studies, coculture setups involving primary neurons, microglia, astrocytes, and oligodendrocytes could indeed be integrated in the system to mimic a healthy or a diseased brain environment and to evaluate the response to therapeutic nucleic acid-loaded LNP (or other nanoparticles) designed for the treatment of different neurological disorders.

## Materials and Methods

### Synthesis and Characterization of the Lipid Nanoparticles

The different LNP formulations, namely, Empty LNP, LNP assembled with the red fluorescent Rhodamine B tagged 18:1 Liss Rhod PE lipid chains (RhB-LNP), and LNP assembled with the 18:1 Liss Rhod PE lipid chains carrying a green fluorescent (6-FAM carboxyfluorescein at the 5′ end of the) scrambled RNA (FAM-RNA-LNP), were all prepared using the NanoAssemblr microfluidic mixer, equipped with a microfluidic mixing cartridge with a staggered herringbone architecture (Precision NanoSystems Inc., San Francisco, USA). A 40 mM lipid mixture in 100% EtOH was mixed with citrate buffer (50 mM, pH 3.9) at a flow rate ratio (FRR) of 1:3 and a total flow rate of 8 mL/min (see Supporting Information Table S1 for LNP material specifications and Supporting Information Table S2 for the molar ratios of each lipid mixture). For FAM-RNA-LNP, scrambled FAM-RNA was dissolved in citrate buffer at a final concentration of 240 μg/mL and mixed with the lipid mixture at a final N/P ratio of 10. Subsequently, LNP were dialyzed against PBS for 20 h at RT and stored at 4 °C. The cutoff/membrane was 3.5 kDa/Pur-A-Lyzer for Empty LNP and RhB-LNP and 300 kDa/Float-A-Lyzer for FAM-RNA-LNP (Sigma-Aldrich, Merck KGaA, Germany) to remove nonencapsulated RNA. The hydrodynamic diameter, zeta potential, and polydispersion index of LNP were assessed by dynamic light scattering (DLS; Zetasizer Nano ZS; Zetasizer software version 8.01.4906, 2002–2020; Malvern Panalytical, United Kingdom) upon 100× dilution in ddH_2_O.

The lipid concentration in Empty LNP was assessed by a modified version of the Stewart assay, which allows colorimetric determination of phospholipids upon the generation of a complex with ammonium ferrothiocyanate. Starting from a stock solution of DOPC (2 mg/mL in 100% EtOH), a calibration curve was generated within the concentration range 0–1 mg/mL. Empty LNP were equally diluted up to 50 μL in ethanol. 950 μL of chloroform and 1 mL of Stewart reagent (27.03 g of ferric chloride FeCl_3_ × 6H_2_O and 30.4 g of ammonium thiocyanate [NH_4_]^+^[SCN]^−^ in 1 L of ddH_2_O) were added to the lipid solutions, then mixed for 20 s, and centrifuged for 10 min at 1000 rpm. The 485 nm absorbance of the halogenated solutions was measured by UV–vis spectrophotometry. A linear least-squares regression was used to fit the data, which returned the function (*R* ^2^ = 0.9963). The 18:1 Liss Rhod PE concentration in the LNP was estimated by fluorescence analysis. A calibration curve was generated in the range of 1 - 3000 ng/mL by diluting a 10 mg/mL stock solution of 18:1 Liss Rhod PE in EtOH. The 18:1 Liss Rhod PE content in LNP was assessed by diluting 1:20 (v/v) the particle dispersion in EtOH and analyzing the fluorescence intensity (excitation/emission: 520/580 nm). A linear least-squares regression was used to fit the data, which returned the function (*R* ^2^ = 0.9919). Finally, the FAM-RNA concentration in the LNP was assessed by fluorescence analysis upon LNP lysis. LNP were lysed in 1% Triton X-100/ddH_2_O and vortexed for 40 min at 600 rpm and 45 °C. A stock solution of FAM-RNA (100 μM in RNase free water) was diluted with a 1% Triton X-100/ddH_2_O solution to prepare a calibration curve within the concentration range 0.01–3 μM. Fluorescence intensity was assessed at the excitation/emission wavelengths of 445/520 nm. A linear least-squares regression was used to fit the data, which returned the function (*R* ^2^ = 0.9974). All absorbance and fluorescence measurements mentioned above were performed on a Tecan Spark plate reader (Tecan Group Ltd., Switzerland) unless differently specified.

### Isolation of Primary Cortical Neurons from Rat Embryos

Pregnant rats were ordered from Charles River Laboratories (Italy). Upon sacrifice of the pregnant rat, the placenta was immediately placed in Hanks’ balanced salt solution (HBSS, #14170088). On embryonic day E17, embryos were detached and decapitated with scissors, and brains were displaced from the skulls with surgical tweezers and placed in cold HBSS. After dividing the two hemispheres, the meninges, the corpus striatum, and the hippocampus were discarded to obtain the cortices. The dissected tissue was then incubated from 30 to 45 min in a digestion solution −1.3 mL/cortex (0.125% Trypsin, #25050014; 25 μg DNase #D5025 in 5 mM CaCl_2_/HBSS per mL of medium). Complete neurobasal medium (NB, #21103049, supplemented with 1% penicillin/streptomycin, P/S, #P4333; 1% GlutaMAX, #35050038; 2% B-27, #17504044) with 10% heat-inactivated horse serum (HS, #26050088) was added to inactivate trypsin prior to 5 min centrifugation at 1200 rpm. Supernatant was then discarded, and fresh 10% HS NB was added before dissociating tissue through gentle pipetting. Upon being filtered through a 40 μm cell strainer, cells were centrifuged again for 7 min at 700 rpm, counted, and resuspended in complete NB before plating at the desired density. Every 3–7 days, 50% of cell culture medium was replaced with fresh complete NB. Cells were cultured and incubated at 37 °C, 5% CO_2_. Any plating surface was previously coated with 0.01% poly- *d* -lysine/ddH_2_O (PDL, #P6407), incubated at 37 °C, 5% CO_2_ for 1 h (for the microfluidic chips)–24 h (for the regular culture plates) and washed 2× with ddH_2_O prior to plating. To perform flow cytometry or microscopy analysis of the LNP cellular uptake, cortical neurons were seeded in poly- *d* -lysinated 12-well plates, at a density of 1.5 × 10^5^/well, either without or with a 12Ø coverslip (sterilized and added prior to PDL coating) in each well, and maintained in 1.5 mL of NB. To perform MTT assay, neurons were seeded at a density of 5 × 10^4^/well in PDL-coated 96-well plates. HBSS, Neurobasal, Glutamax, B-27, and HS were purchased from Gibco, Thermo Fisher Scientific, MA, USA. Trypsin, DNase, P/S, and PDL were purchased from Sigma-Aldrich, Merck KGaA, Germany. Plates were purchased from Corning, NY, USA.

### MTT Assay on Cortical Neurons upon LNP Treatment

Cortical neurons were incubated with Empty LNP (0, 1, 5, 10, 50, 100 μg lipids/mL of NB medium) for 2, 24, and 48 h. Culture medium was then replaced with 0.25 mg/mL MTT/NB medium (3-(4,5-dimethyl-2-thiazolyl)-2,5-diphenyl-2H-tetrazolium bromide, #475989, Sigma-Aldrich, Merck KGaA, Germany) for 4 h. Subsequently, MTT was removed and replaced with 100% EtOH and the absorbance of MTT-formazan product read at 570 nm at the spectrophotometer (Tecan Spark, Tecan Group Ltd., Switzerland).

### Flow Cytometry of Cortical Neurons upon LNP Treatment

Cortical neurons were incubated with RhB-LNP and FAM-RNA-LNP (5 μg of lipids/mL of NB medium) for 0.5, 1, or 2 h. Cells were then washed with PBS and given 100 μL of Trypsin/EDTA for 5 min. Cells were then gently resuspended by pipetting after 200 μL of 10% HS NB was added and filtered through 70 μm cell strainers to avoid aggregates. Cells were then kept on ice and sorted with a BD FACSAria II flow cytometer (Becton, Dickinson BD Biosciences, NJ, USA). Considering the absorbance spectrum of each fluorescent molecule, namely, lissamine rhodamine B PE and FAM, the phycoerythrin laser setting was used to measure the RhB-lipid signal while the FITC laser setting was used for the FAM-RNA signal. The PE intensity was used to set the gating to measure the percentage of cells positive for the uptake of both RhB- and FAM-RNA-LNP, before assessing the median cellular intensity of the two fluorophores (RhB and FAM) in the positive cells. The main population was gated as P1 and results on the cellular intensity exported to calculate the LNP uptake based on RhB-lipid and FAM-RNA fluorescence associated with cells. PBS and Trypsin/EDTA were purchased from Sigma-Aldrich, Merck KGaA, Germany.

### Immunostaining of Cortical Neurons upon LNP Treatment

Cortical neurons were incubated with FAM-RNA-LNP (5 μg of lipids/mL of NB medium) for 0.5, 1, 2, 4, 6, and 8 h. Cells were washed with PBS and then fixed in 4% paraformaldehyde for 15 min at RT, washed 2× with PBS, and stored at 4 °C. For immunostaining, cells were then permeabilized for 15 min with 0.7% Triton X-100 (#T8787, Sigma-Aldrich, Merck KGaA, Germany), washed 2× with PBS, and blocked 30 min with 1% BSA/PBS (bovine serum albumin, #A4503, Sigma-Aldrich, Merck KGaA, Germany). Upon blocking, cells were incubated for 1 h at RT with antibodies to label the neuronal marker neurofilament heavy, NF200 (primary mouse IgG1 anti-NF200 monoclonal antibody—clone NE14, #N5389, Sigma-Aldrich, Merck KGaA, Germany; secondary goat anti-mouse IgG1 AF647 antibody, #A-21240, Thermo Fisher Scientific, Waltham, MA, USA; 1:800 dilution in 1% BSA/PBS). Before and after the incubation with the secondary antibody, cells were washed 2 × 5 min with 1× PBS and 2 × 10 min with 0.01% Tween 20 (#9127.1, Carl Roth, Karlsruhe, Germany). All dilutions mentioned above were done in 1× PBS, except antibody dilutions in 1% BSA/1× PBS. DNA was counterstained with 10 μg/mL DAPI (4′,6-diamidino-2-phenylindole, #D27802, Sigma-Aldrich, Merck KGaA, Germany) for 10 min at RT, cells dipped in ddH_2_O, and mounted with ProLong Gold Antifade Mountant (#P36930, Invitrogen, Thermo Fisher).

### Confocal Imaging

Samples of cortical neurons on coverslips, incubated with FAM-RNA-LNP as described above, were imaged with a Nikon Ti Eclipse A1 laser scanning confocal microscope (scanner Galvo Mirror, detector 4 PMT DU4, Nikon Instruments Inc., NY, USA) with a 60× oil objective (Plan Apo λ NA 1.4) and laser wavelengths 405, 488, 561, and 640 to image DNA, FAM, RhB, and the neurofilament heavy NF200, respectively. Images were acquired using the software NIS-Elements AR 4.20.00 (Build 967; NIKON Corporation 1991–2013, Laboratory Imaging).

### Culture and Treatment of Cortical Neurons in Microfluidic Chips

Microfluidic chips with two distinct compartments separated by 150 μm-long microgrooves (XonaChip, #XC150, Xona Microfluidics, USA) were prepared according to the manufacturer’s instructions. Specifically, each chip was first treated with XC Pre-Coat (Xona Microfluidics), then washed 2× with PBS, incubated with 0.01% PDL for 1 h at 37 °C and 5% CO_2_, and washed 2× with ddH_2_O before adding complete NB. Each solution was added as follows to allow proper fluid movement without bubble formation: 150 μL was added in the upper left well; after 1 min, 150 μL was added in the lower left well; after 5 min, 150 μL was added in the upper right well; and after 1 min, 150 μL was added in the lower right well. Unless specified, the subsequent solution/washing was performed after 5 min. Once cortical neurons were isolated, NB was removed from the chips, and 5 μL of a 12 million/mL cell suspension in NB was injected into the channel from both reservoirs on one side of the chip (10 μL in total), which hereafter will be referred to as the “somal compartment”. Complete NB was then given to both upper wells and subsequently to both lower wells. Before LNP treatment, old medium was replaced from the wells with 150 μL of fresh NB per well, without removing the medium from the microgrooves. Fluidic isolation of the somal compartment from the axonal one was performed by applying a volume difference of 20 μL between the two sides of the chip. After 10 min, FAM-RNA-LNP were injected in the axonal compartment through both the corresponding wells and incubated 4 h before time-lapse imaging until 8 h of treatment.

### Time-Lapse Imaging

Samples of cortical neurons on a chip, incubated with FAM-RNA-LNP as described above, were imaged at a wide-field fluorescence microscope Nikon Ti Eclipse (camera Andor DU-89; detector ANDOR iXon; light source cool LED pE-300^ultra^; transmitted light Ti Illuminator-DIA; Nikon Instruments Inc., NY, USA) with a 20× air objective (Plan Apo VC DIC N2 NA 0.8). The following setup of filters and dichroic mirrors was used: EX400-440, DM455, BA470 for FAM-RNA; EX540/25, DM565, BA605/55 for RhB. Images were acquired every 5 min for 4 h using the software NIS-Elements AR 5.00.00 (Build 1223; NIKON Corporation © 1991–2017 Laboratory Imaging).

### Image Analysis

Basic image processing and production of all montages for an overview of the samples were done using Fiji software ((Fiji Is Just) ImageJ 2.9.0/1.54f; Java 1.8.0_322; 64 bit). Analyses of the confocal images and time-lapse microscopy images were performed through the software NIS-Elements AR Analysis (version 4.20.03 (Build 995); NIKON Corporation, © 1991–2016 Laboratory Imaging). For the confocal images, 32 different 10 μm × 10 μm regions of interest (ROI) were selected in the three most central *z*-planes of each confocal image stack (16 located on the soma and 16 on axons, based on the neurofilament signal). The sum intensity in these ROI was extracted for both the RhB and FAM channels. Within these ROI, the NIS plugin Colocalization was run to obtain the Pearson correlation coefficient and the Manders’ overlap coefficient. For the time-lapse microscopy images, the whole somal compartment of different fields of view for each microfluidic chip was cropped; the values of mean intensity and related standard deviation for the RhB-lipid and FAM-RNA signals were calculated and normalized to the time point 0 of imaging, corresponding to 4 h of LNP treatment, to produce violin plots. Additionally, a threshold-based mask in the FAM channel was applied to identify single neurons throughout the whole temporal stack; the number, the related intensity, and occupied area of single neurons which become over-the-threshold positive over time were extracted.

### Data Visualization and Statistics

All experimental results were processed for statistical analysis and data plotting with RStudio (Version 0.99.902 © 2009–2016 RStudio Inc., Boston, United States). See Supporting Information Table S3 for all statistical analyses and Supporting Information Figure S8 for boxplot and violin plot interpretation.

## Supplementary Material



## Notes

Material for LNP production, lipid’s molar ratio in LNP, statistical significance; additional data and methods on LNP characterization, neuronal uptake and image analysis pipelines (PDF)
