# Supplementary material for: On The Retrograde Transport of RNA-Loaded Lipid Nanoparticles Designed for Brain Delivery
Source: ACS Nanosci Au. 2025 Aug 22;5(5):375–87. doi: 10.1021/acsnanoscienceau.5c00042 (PMC12531864; doi:10.1021/acsnanoscienceau.5c00042)
Supplement: Supplementary file 1 [file ng5c00042_si_001.pdf]

ON THE RETROGRADE TRANSPORT OF RNA LOADED LIPID NANOPARTICLES DESIGNED FOR BRAIN DELIVERY

Stefania Mamberti[1], Cristiano Pesce[1,2], Greta Avancini[1], Gonna Somu Naidu[3-6], Govinda Reddy Kundoor[3-6], Corinne Portioli[1], Dan Peer[3-6], Paolo Decuzzi[1,7], Roberto Palomba[1 *]

[1] Laboratory of Nanotechnology for Precision Medicine, Italian Institute of Technology, Genoa 16163, Italy.

[2] Department of Pharmaceutical and Pharmacological Sciences, University of Padua, Padua 35131, Italy.

[3] Laboratory of Precision Nanomedicine, Shmunis School of Biomedicine and Cancer Research, Tel Aviv University, Tel Aviv-Yafo 69978, Israel.

[4] Department of Materials Sciences and Engineering, Tel Aviv University, Tel Aviv-Yafo 69978, Israel.

[5] Center for Nanoscience and Nanotechnology, Tel Aviv University, Tel Aviv-Yafo 69978, Israel.

[6] Cancer Biology Research Center, Tel Aviv University, Tel Aviv-Yafo 69978, Israel.

[7] School of Medicine/Division of Oncology, Center for Clinical Sciences Research, Stanford University, Stanford 94305, USA.

[*] corresponding author: Dr. Roberto Palomba, roberto.palomba@unina.it

SUPPLEMENTARY TABLES, FIGURES AND TEXT

List of contents:

Supplementary Table S1: Material for LNP production.

Supplementary Table S2: Lipid content within each LNP formulation.

Supplementary Table S3: Statistical significance.

Supplementary Figure S1: LNP lyophilization studies.

Supplementary Figure S2: LNP biocompatibility in primary cortical neurons.

Supplementary Figure S3: Flow cytometry gating strategy for LNP uptake in neurons.

Supplementary Figure S4: Manders' and Pearson's coefficients in somal and axonal regions.

Supplementary Figure S5: Image analysis pipeline for threshold-based object identification.

Supplementary Figure S6: Timeline overview of FAM-RNA cargo release in single neurons.

Supplementary Figure S7: Quantification of FAM-RNA cargo release in single neurons.

Supplementary Figure S8: Boxplot and violin plot interpretation.

Supplementary Material and Methods.

Supplementary References.

Supplementary Table S1: Material for LNP production.

Material		Abbreviation	Nomenclature in Figure 1A	Source
**Lipids**	1,2-dioleoyl-sn-glycero-3-phosphoethanolamine-N-(lissamine rhodamine B sulfonyl) (ammonium salt)	18:1 Liss Rhod PE	**Rhodamine B lipid**	Avanti Polar, Alabaster, AL, USA
	8-((2-((4-(dimethylamino)butanoyl)oxy)ethyl)((9Z,12Z)-octadeca-9,12-dien-1-yl)amino)octyl 2-hexyldecanoate	Lipid 15	**Ionizable lipid**	Dan Peer's Laboratory (Tel Aviv University), lipid #15 in [1].
	Cholesterol	Cholesterol	**Cholesterol**	Sigma-Aldrich®, Merck KGaA, Germany
	1,2-dioleoyl-sn-glycero-3-phosphocholine	DOPC	**Helper lipid**	Avanti Polar, Alabaster, AL, USA
	1,2-dimyristoyl-rac-glycero-3-methoxypolyethylene glycol-2000	DMG-PEG	**PEGylated lipid**	Avanti Polar, Alabaster, AL, USA
**Nucleic acid**	5'-/6-FAM/mU*mC*mG*mUmUmAmAmUmCmGmGmCmUmAmUmAmAmUmA*mC*mG*mC-3'	Fluorescent-scrambled RNA	**FAM-RNA**	Integrated DNA Technologies, Iowa, USA

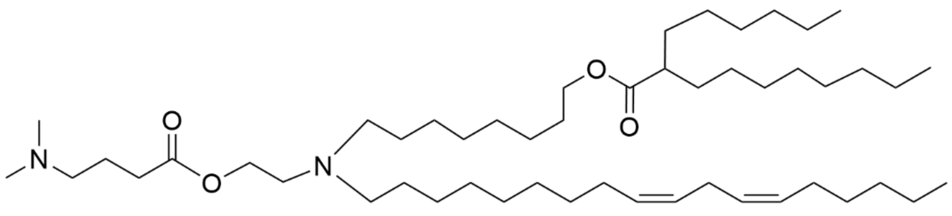

Lipid 15

Supplementary Table S2: Lipid content within each LNP formulation.

<table>
<tr><th></th><th>Empty LNP</th><th>RhB-LNP</th><th>FAM-RNA-LNP</th></tr>
<tr><td>Lipids</td><td colspan="3">molar ratios of the lipid mixture in 100% EtOH</td></tr>
<tr><td>Lipid 15 (Ionizable lipid)</td><td>50</td><td colspan="2">50</td></tr>
<tr><td>Cholesterol</td><td>38</td><td colspan="2">38</td></tr>
<tr><td>DOPC</td><td>10.5</td><td colspan="2">10.4</td></tr>
<tr><td>DMG-PEG</td><td>1.5</td><td colspan="2">1.5</td></tr>
<tr><td>18:1 Liss Rhod PE</td><td>-</td><td colspan="2">0.1</td></tr>
</table>

Supplementary Table S3: Statistical significance.

Figure	Parameter	Samples tested		p-value
1B	Size (nm)	Empty LNP	RhB-LNP	1
		Empty LNP	FAM-RNA-LNP	0.2
		FAM-RNA-LNP	RhB-LNP	0.2
	ζ-potential (mV)	Empty LNP	RhB-LNP	0.7
		Empty LNP	FAM-RNA-LNP	0.1
		FAM-RNA-LNP	RhB-LNP	0.1
1E	Percentage of positive cells (%)	RhB-LNP × 0.5 h	FAM-RNA-LNP × 0.5 h	0.2
		RhB-LNP × 1 h	FAM-RNA-LNP × 1 h	0.2
		RhB-LNP × 2 h	FAM-RNA-LNP × 2 h	0.1
2C	RhB-lipid sum intensity (au.)	FAM-RNA-LNP × 0.5 h	FAM-RNA-LNP × 1 h	< 2.2e-16
		FAM-RNA-LNP × 0.5 h	FAM-RNA-LNP × 2 h	< 2.2e-16
		FAM-RNA-LNP × 0.5 h	FAM-RNA-LNP × 4 h	< 2.2e-16
		FAM-RNA-LNP × 0.5 h	FAM-RNA-LNP × 6 h	< 2.2e-16
		FAM-RNA-LNP × 0.5 h	FAM-RNA-LNP × 8 h	< 2.2e-16
2D	FAM-RNA sum intensity (a.u.)	FAM-RNA-LNP × 0.5 h	FAM-RNA-LNP × 1 h	0.001411
		FAM-RNA-LNP × 0.5 h	FAM-RNA-LNP × 2 h	1.79e-06
		FAM-RNA-LNP × 0.5 h	FAM-RNA-LNP × 4 h	< 2.2e-16
		FAM-RNA-LNP × 0.5 h	FAM-RNA-LNP × 6 h	< 2.2e-16
		FAM-RNA-LNP × 0.5 h	FAM-RNA-LNP × 8 h	< 2.2e-16
2E		FAM-RNA-LNP × 0.5 h	FAM-RNA-LNP × 1 h	2.246e-12

	**Manders' colocalization overlap (a.u.)**	FAM-RNA-LNP × 0.5 h	FAM-RNA-LNP × 2 h	< 2.2e-16
		FAM-RNA-LNP × 0.5 h	FAM-RNA-LNP × 4 h	< 2.2e-16
		FAM-RNA-LNP × 0.5 h	FAM-RNA-LNP × 6 h	< 2.2e-16
		FAM-RNA-LNP × 0.5 h	FAM-RNA-LNP × 8 h	< 2.2e-16
**2F**	**Pearson's correlation coefficient (a.u.)**	FAM-RNA-LNP × 0.5 h	FAM-RNA-LNP × 1 h	2.454e-07
		FAM-RNA-LNP × 0.5 h	FAM-RNA-LNP × 2 h	< 2.2e-16
		FAM-RNA-LNP × 0.5 h	FAM-RNA-LNP × 4 h	< 2.2e-16
		FAM-RNA-LNP × 0.5 h	FAM-RNA-LNP × 6 h	< 2.2e-16
		FAM-RNA-LNP × 0.5 h	FAM-RNA-LNP × 8 h	< 2.2e-16
**3C**	**Axonal growth length (µm)**	Day 4	Day 5	6.386e-05
		Day 5	Day 6	0.01854
		Day 6	Day 7	0.3527
**4C**	**RhB Mean (a.u.)**	FAM-RNA-LNP × 4 h	FAM-RNA-LNP × 5 h	6.777e-07
		FAM-RNA-LNP × 4 h	FAM-RNA-LNP × 6 h	6.777e-07
		FAM-RNA-LNP × 4 h	FAM-RNA-LNP × 7 h	6.777e-07
		FAM-RNA-LNP × 4 h	FAM-RNA-LNP × 8 h	6.777e-07
	**RhB StDev (a.u.)**	FAM-RNA-LNP × 4 h	FAM-RNA-LNP × 5 h	0.002961
		FAM-RNA-LNP × 4 h	FAM-RNA-LNP × 6 h	6.777e-07
		FAM-RNA-LNP × 4 h	FAM-RNA-LNP × 7 h	6.777e-07
		FAM-RNA-LNP × 4 h	FAM-RNA-LNP × 8 h	6.777e-07
		FAM-RNA-LNP × 4 h	FAM-RNA-LNP × 5 h	0.1008

	**FAM Mean (a.u.)**	FAM-RNA-LNP × 4 h	FAM-RNA-LNP × 6 h	6.777e-07
		FAM-RNA-LNP × 4 h	FAM-RNA-LNP × 7 h	6.777e-07
		FAM-RNA-LNP × 4 h	FAM-RNA-LNP × 8 h	6.777e-07
	**FAM StDev (a.u.)**	FAM-RNA-LNP × 4 h	FAM-RNA-LNP × 5 h	6.866e-07
		FAM-RNA-LNP × 4 h	FAM-RNA-LNP × 6 h	0.7563
		FAM-RNA-LNP × 4 h	FAM-RNA-LNP × 7 h	0.0002778
		FAM-RNA-LNP × 4 h	FAM-RNA-LNP × 8 h	0.0002778
**5B**	**FAM-RNA Sum intensity in single neurons (a.u.)**	FAM-RNA-LNP × 5 h	FAM-RNA-LNP × 6 h	0.01439
		FAM-RNA-LNP × 5 h	FAM-RNA-LNP × 7 h	0.003959
		FAM-RNA-LNP × 5 h	FAM-RNA-LNP × 8 h	0.001164
	**FAM-RNA-positive area of single neurons (px^2)**	FAM-RNA-LNP × 5 h	FAM-RNA-LNP × 6 h	0.01107
		FAM-RNA-LNP × 5 h	FAM-RNA-LNP × 7 h	0.003448
		FAM-RNA-LNP × 5 h	FAM-RNA-LNP × 8 h	0.0007743
**Suppl. 2**	**Cell viability (%)**	0.0× empty LNP × 2 h	0.1× empty LNP × 2 h	0.1066
		0.0× empty LNP × 2 h	0.5× empty LNP × 2 h	1
		0.0× empty LNP × 2 h	1× empty LNP × 2 h	0.6428
		0.0× empty LNP × 2 h	5× empty LNP × 2 h	0.6428
		0.0× empty LNP × 2 h	10× empty LNP × 2 h	0.6428
		0.0× empty LNP × 24 h	0.1× empty LNP × 24 h	0.1066
		0.0× empty LNP × 24 h	0.5× empty LNP × 24 h	0.6428
		0.0× empty LNP × 24 h	1× empty LNP × 24 h	0.6428
		0.0× empty LNP × 24 h	5× empty LNP × 24 h	1

		0.0× empty LNP × 24 h	10× empty LNP × 24 h	0.6428
		0.0× empty LNP × 48 h	0.1× empty LNP × 48 h	0.0636
		0.0× empty LNP × 48 h	0.5× empty LNP × 48 h	1
		0.0× empty LNP × 48 h	1× empty LNP × 48 h	0.6428
		0.0× empty LNP × 48 h	5× empty LNP × 48 h	1
		0.0× empty LNP × 48 h	10× empty LNP × 48 h	0.2482

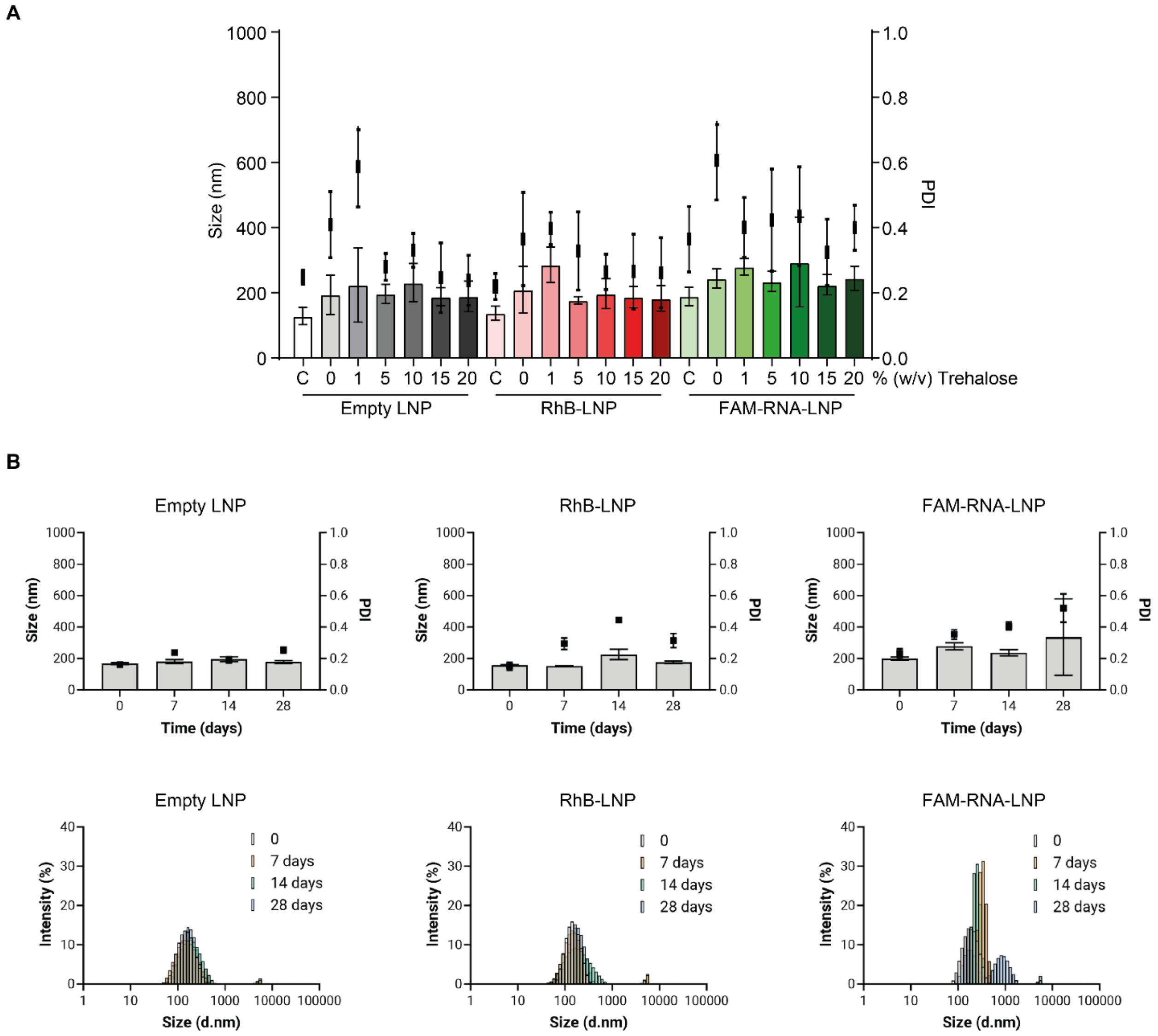

Supplementary Figure S1: LNP lyophilization studies.

A. Dynamic light scattering measurements of Empty, RhB- and FAM-RNA-LNP upon lyophilization. The three formulations were freeze-dried with different percentages of trehalose (0, 1, 5, 10, 15, 20 % w/v). Size and PDI after lyophilization and subsequent resuspension in ddH_2O are compared with the respective fresh LNP sample ("C" in the plot). **B.** Stability of the formulations lyophilized with 15 % (w/v) trehalose over time. Size and PDI after lyophilization and subsequent storage for 0, 7, 14 and up to 28 days, together with the related intensity (%), whose distribution shows the consistency of the measurements over time.

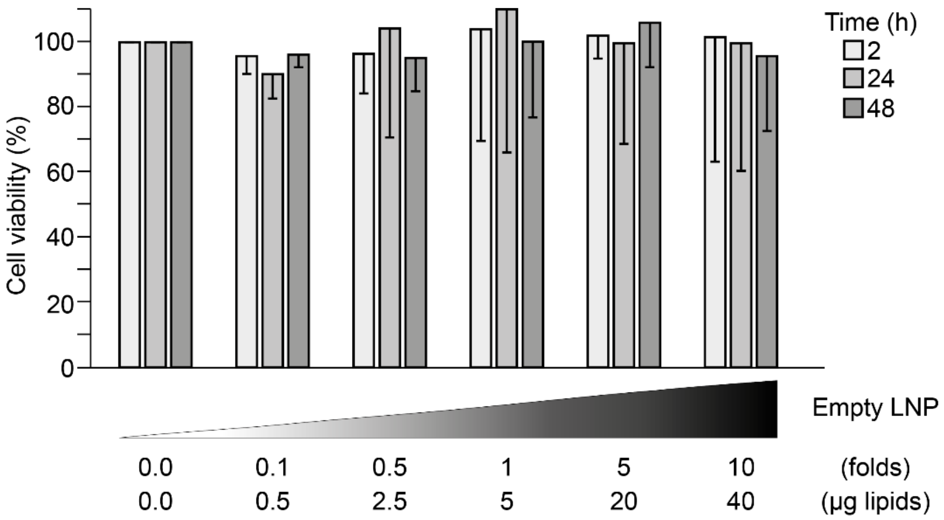

Supplementary Figure S2: LNP biocompatibility in primary cortical neurons.

Biocompatibility was assessed by incubating different amounts of Empty LNP (0 to 40 µg of total lipid content) with primary cortical neurons and measuring the metabolic activity at 2, 24 and 48 h via the MTT assay.

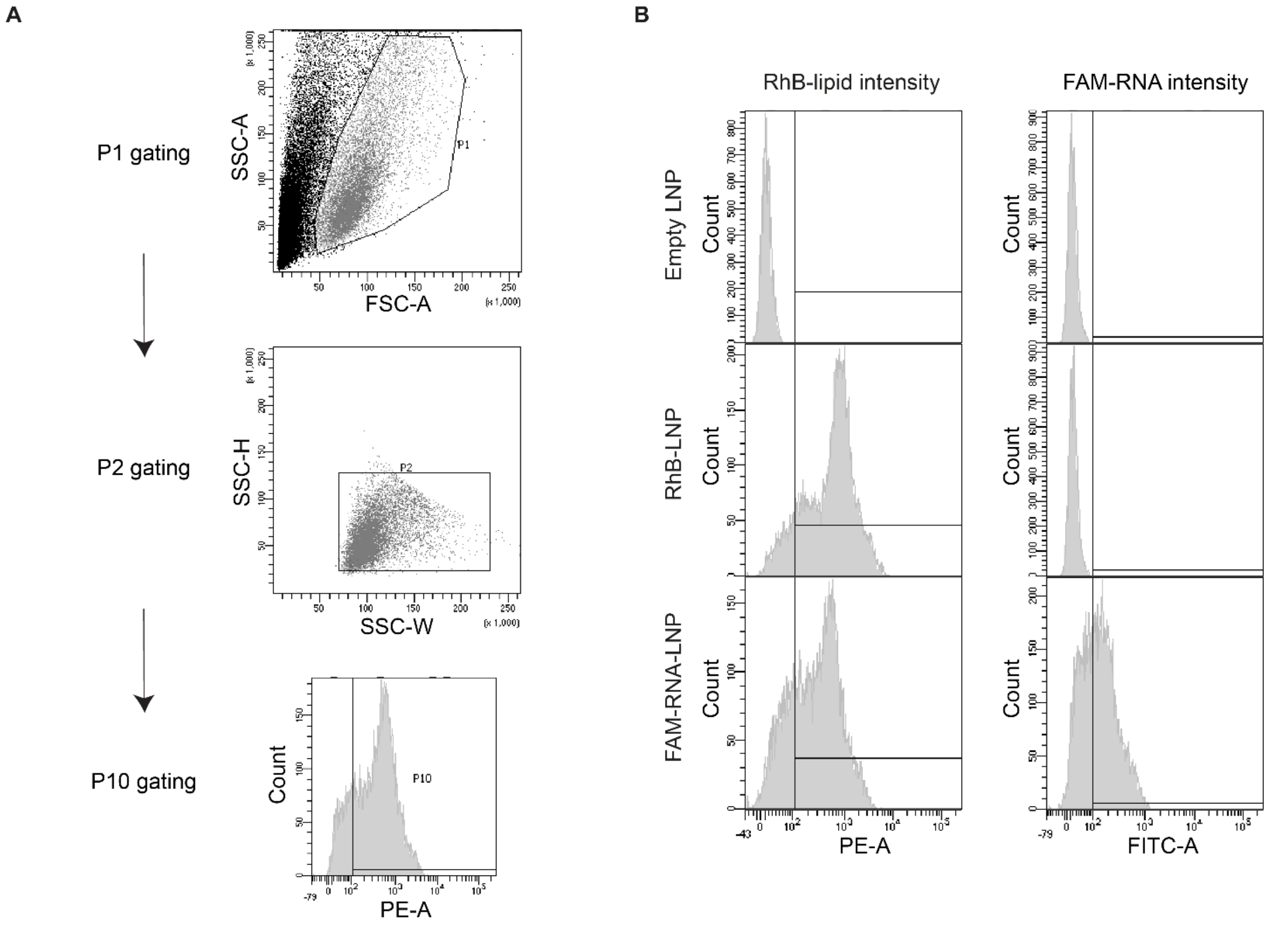

Supplementary Figure S3: Flow cytometry gating strategy for LNP uptake in neurons.

A. Representative images of the gating pipeline used in the flow cytometry analyses of neuronal LNP uptake. The cortical neurons population was first selected as P1 based on the Forward Scatter Area (FSC-A) and Side Scatter Area (SSC-A) parameters, which respectively represent the cellular size and the intracellular complexity or granularity, allowing the exclusion of cellular debris. From the P1 population, the P2 gating was applied based on the Side Scatter Height (SSC-H) and Side Scatter Width (SSC-W) to further exclude extreme values of granularity. From the P2 population, the P10 gate was derived by setting a threshold on the fluorescence intensity measured with the phycoerythrin laser (PE-A) at 10^2 a.u., value at which the cells treated with Empty LNP did not show any signal. The resulting P10 population was considered as the neuronal population positive for interaction with LNP due to the detection of the RhB-lipid (percentage of positive cells) and used to extract the RhB-lipid and FAM-RNA cellular median intensity values.

B. Representative images of the measures of cellular fluorescence intensity in the flow cytometry analyses for neurons incubated with Empty LNP, RhB-LNP, and FAM-RNA-LNP for 2 hours. The phycoerythrin laser (PE-A) was used to measure the RhB-lipid signal while the FITC laser (FITC-A) was used for the FAM-RNA signal. The median fluorescence intensity was extracted from the P10 population gated based on the PE-A threshold for both RhB-LNP and FAM-RNA-LNP treatments.

A

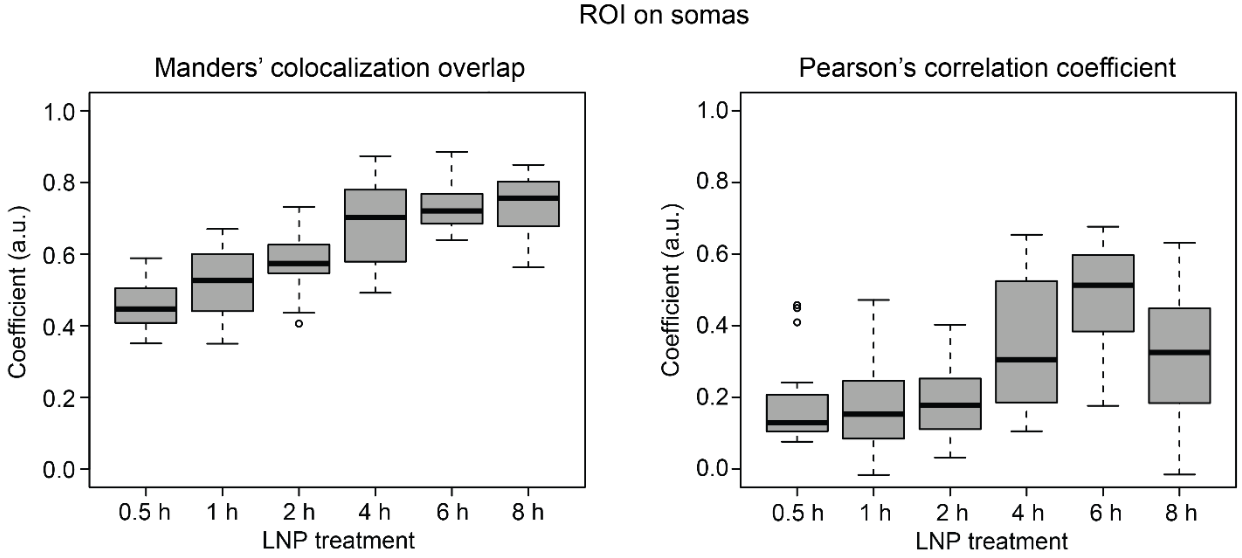

B

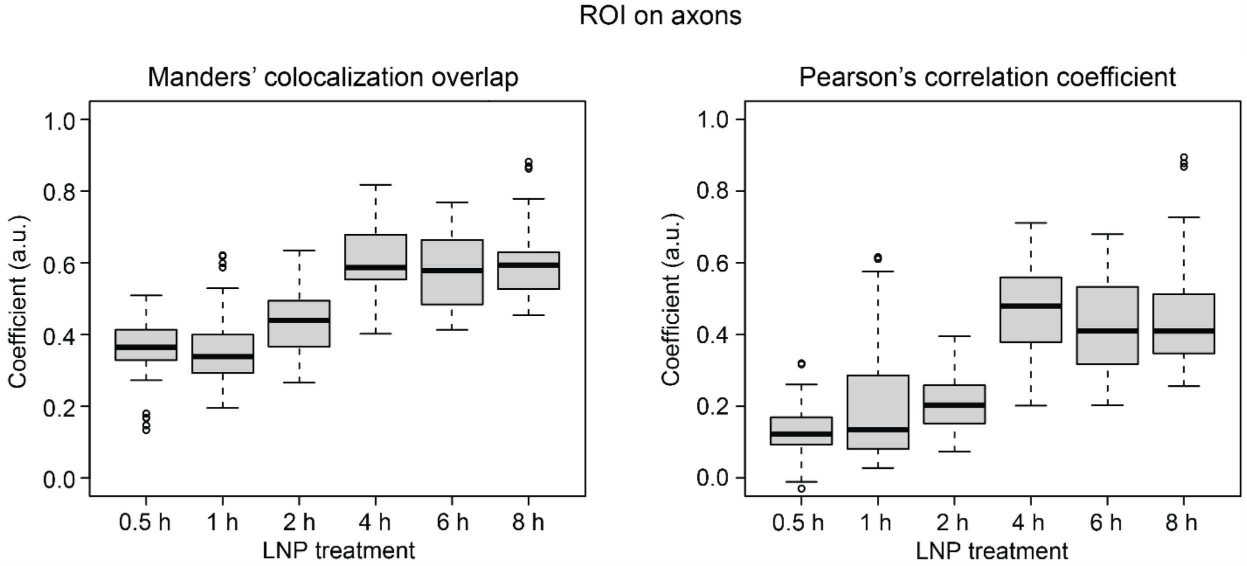

Supplementary Figure S4: Manders' and Pearson's coefficients in somal and axonal regions.

A. Manders' colocalization overlap and Pearson's correlation coefficient for ROI located on the cellular bodies or somas of cortical neurons. **B.** Manders' colocalization overlap and Pearson's correlation coefficient for ROI located on the axons of cortical neurons.

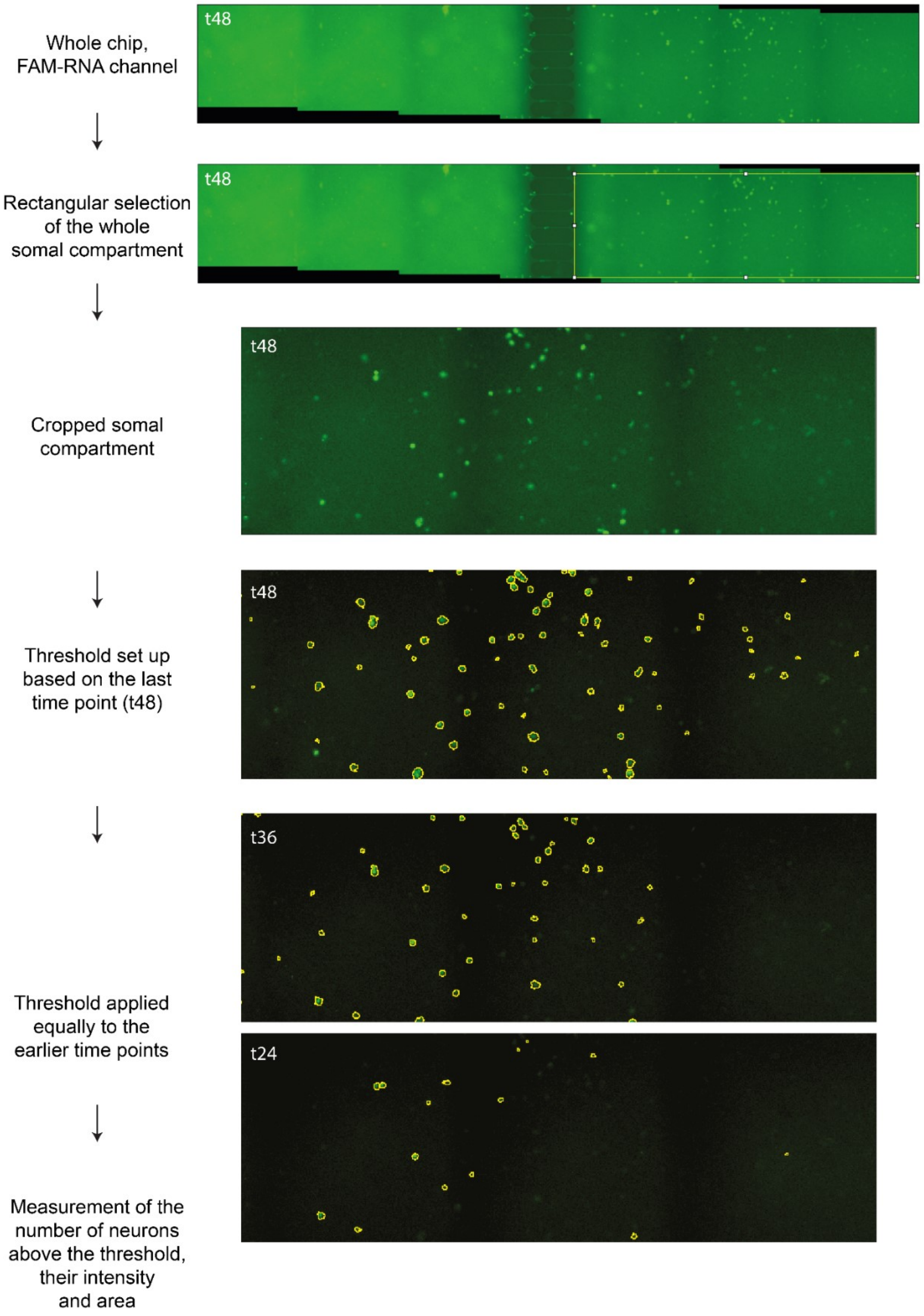

Supplementary Figure S5: Image analysis pipeline for threshold-based object identification.

In order to consistently identify the cellular bodies of cortical neurons showing FAM-RNA accumulation, a threshold was set through image analysis. First, the somal compartment was cropped out from the mosaic of images corresponding to the whole chip. Then, a threshold for the FAM signal

intensity was set by using the last time frame (i.e. t48) of the microscopy recordings, so that all FAM-RNA-enriched somas would result selected (segmented). The same threshold was then equally applied “backwards” in time to the earlier time points (e.g. t36, t24). This strategy allowed us to finely tune the threshold to select the positive neurons while tackling possible issues of debris or background selection.

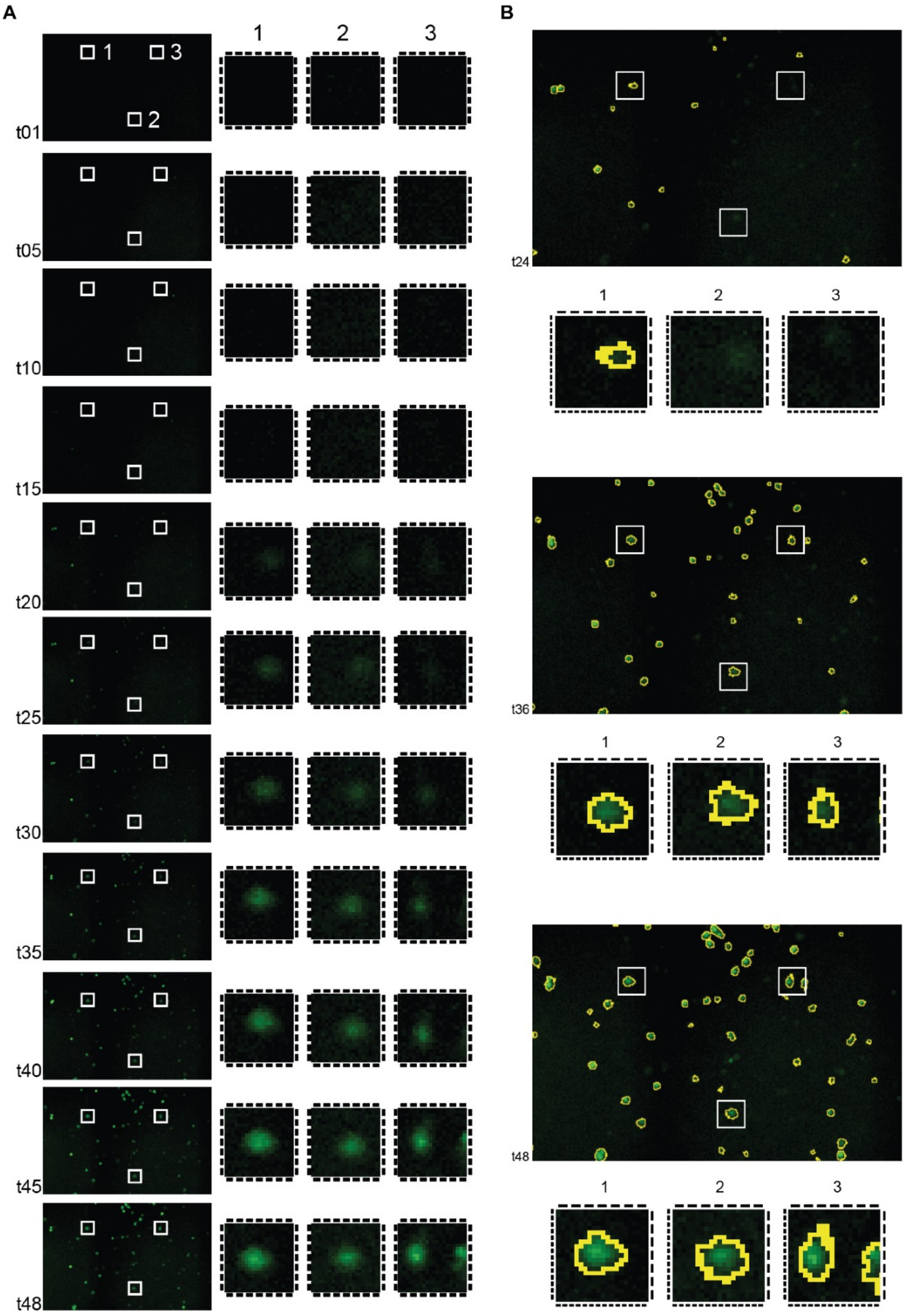

Supplementary Figure S6: Timeline overview of FAM-RNA cargo release in single neurons.

A. Raw time lapse microscopy images of FAM-RNA accumulation in somal bodies. **B.** Image analysis based segmentation of FAM-RNA-positive somal bodies. In both panels, **A** and **B**, selected time frames acquired in the FAM-RNA channels are shown (t01 - t48; where t01 = 4 h, t12 = 5 h, t24 = 6 h, t36 = 7 h, t48 = 8 h of FAM-RNA-LNP incubation). Three areas, (1, 2, 3), are selected from the frame (white squares) and enlarged close to the corrisponding frame (dashed black squares) to show the gradual enlightnening of the cellular bodies (FAM signal gradual increase, in **A**) and the resulting selection of these bodies as FAM-RNA-positive neurons (segmentation upon threshold-based image analysis, in **B**).

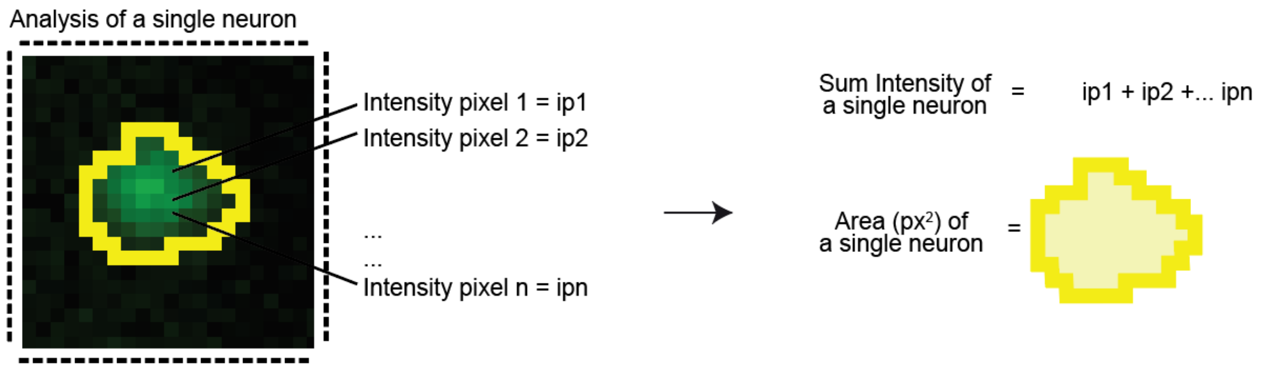

Supplementary Figure S7: Quantification of FAM-RNA cargo release in single neurons.

The figure in the dashed square on the left shows the magnification of one of the neuronal bodies (somas) that were detected for being positive to FAM-RNA signal through image analysis, as described in the main text relative to **Figure 5**. Briefly, a threshold was set based on FAM intensity signal to segment single somas. The yellow border defines the area selected based on the intensity threshold. This area increases when more pixels get to an intensity that is higher than the threshold. What is referred to in the main text as the FAM-RNA sum intensity in single neurons is the sum of the intensity of all the pixels contained in the so defined area.

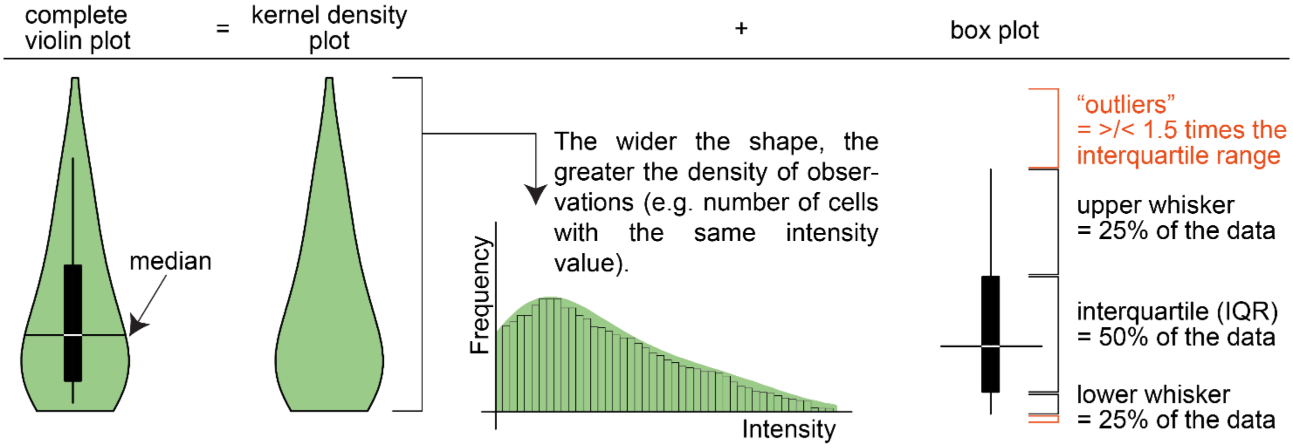

Supplementary Figure S8: Boxplot and violin plot interpretation.

The violin plot is composed of a kernel density plot and a boxplot. The kernel density plot describes the frequency of observations for defined values of the measured parameter. A higher number of observations at a defined value determines a wider shape in correspondence of that value, while thinner parts of the violin shape indicate that a fewer number of observations is present at the corresponding values. Boxplots, which can be visualized independently or embedded in violin plots, describe the major statistical values for the measured parameter, i.e. the median, the interquartile (IQR) range within the box and the remaining data along the whiskers, while the data above and below whiskers are considered to be outliers. Boxplots were used to visualize the data in **Figure 2C-F**. Violin plots were used to visualize the data in **Figure 4C** and **Figure 5B**.

Supplementary Material and Methods

Freeze-Drying Studies

LNP were freeze-dried in the presence of trehalose as a cryoprotectant. Increasing volumes of a 250 mg/mL trehalose solution in Milli-Q water were added to 10 μL aliquots of Empty, RhB- or FAM-RNA-LNP aqueous suspensions to achieve the final sugar concentrations of 1, 5, 10, 15 and 20% (w/v). Freeze-dried samples were then resuspended in Milli-Q water at the same concentration as before lyophilization and analyzed by DLS. The intensity-weighted mean value and PDI were recorded for each sample as the average of three measurements. Results were analyzed using ZS XPLORER 2.0.1.1 software.

Supplementary References

(1) Elia, U.; Ramishetti, S.; Rosenfeld, R.; Dammes, N.; Bar-Haim, E.; Naidu, G. S.; Makdasi, E.; Yahalom-Ronen, Y.; Tamir, H.; Paran, N.; Cohen, O.; Peer, D. Design of SARS-CoV-2 HFc-Conjugated Receptor-Binding Domain MRNA Vaccine Delivered via Lipid Nanoparticles. *ACS Nano* **2021**, *15* (6), 9627–9637. DOI: 10.1021/acsnano.0c10180.
